# Moderate High Caloric Maternal Diet Impacts Dam Breast Milk Metabotype and Offspring Lipidome in a Sex-Specific Manner

**DOI:** 10.3390/ijms21155428

**Published:** 2020-07-30

**Authors:** Marie-Cécile Alexandre-Gouabau, Agnès David-Sochard, Anne-Lise Royer, Patricia Parnet, Vincent Paillé

**Affiliations:** 1 Unité Mixte de Recherche 1280, Laboratoire de Physiopathologie des Adaptations Nutritionnelles, Institut National de la Recherche pour l’Agriculture, l’Alimentation et l’Environnement (INRAe), LUNAM Université, Centre Hospitalier Universitaire Hôtel-Dieu, Place Alexis Ricordeau, HNB1, 44093 Nantes CEDEX 1, France; agnes.david@univ-nantes.fr (A.D.-S.); Patricia.Parnet@univ-nantes.fr (P.P.); vincent.paille@univ-nantes.fr (V.P.); 2 USC INRA 1329, Laboratoire d’Etude des Résidus et Contaminants dans les Aliments (LABERCA), LUNAM Université, ONIRIS, F-44000 Nantes, France; anne-lise.royer@oniris-nantes.fr

**Keywords:** DOHaD, milk phenotyping, lactational programming, high caloric challenge, offspring lipidome

## Abstract

Lactation is a critical period during which maternal sub- or over-nutrition affect milk composition and offspring development that can have lasting health effects. The consequences of moderate high-fat, high-simple carbohydrate diet (WD) consumption by rat dams, during gestation and lactation, on milk composition and offspring blood lipidome and its growth, at weaning, were investigated by using a comprehensive lipidomic study on mass-spectrometric platform combined to targeted fatty- and free amino-acids analysis. This holistic approach allowed clear-cut differences in mature milk-lipidomic signature according to maternal diet with a similar content of protein, lactose and leptin. The lower WD-milk content in total fat and triglycerides (TGs), particularly in TGs-with saturated medium-chain, and higher levels in both sphingolipid (SL) and TG species with unsaturated long-chain were associated to a specific offspring blood-lipidome with decreased levels in TGs-containing saturated fatty acid (FA). The sexual-dimorphism in the FA-distribution in TG (higher TGs-rich in oleic and linoleic acids, specifically in males) and SL species (increased levels in very long-chain ceramides, specifically in females) could be associated with some differences that we observed between males and females like a higher total body weight gain in females and an increased preference for fatty taste in males upon weaning.

## 1. Introduction

As the prevalence of overweight and obesity is a growing global health problem [1], the number of infants born to obese mothers has increased accordingly [2,3] with subsequent increased risk of overweight and childhood obesity [4]. In the United States, about 62% of the female population above 20 years of age is overweight and 33% is obese, based on WHO body mass index criteria [5]. Based on the Center for Health and Disease Criteria, i.e., weight for height above two standard deviations for age and gender [6], 11% of American children are considered as obese, and besides that a significant increase in birth weight in various North American and European populations was observed [7]. The increasing prevalence of maternal obesity triggers a vicious circle: Obese women give birth to children who have developed in an altered in utero environment, predisposing them to develop childhood obesity, metabolic syndrome, and diabetes [8]. The best predictor of the risk of obesity at 2 to 4 years of age seems to be the mother’s pre-gravid or early pregnancy body mass index (BMI) [8,9]. In addition, since childhood overweight is a powerful predictor of obesity in adults, early metabolic illness will have serious consequences on adulthood health [10]. The role of genetic versus environmental factors to explain the mechanisms underlying the emergence of metabolic illness is still poorly understood [11]. Indeed, epigenetic modifications involved in the regulation of gene expression can increase the risk of developing obesity and metabolic syndrome since only less than 10% of obesity cases can be related to genetics [12]. Several routes through which the risk of obesity-related alterations could be transmitted from mother to offspring have been proposed in addition to genetic inheritance. Obese mothers, due to greater or unbalanced altered food intake and/or obesity per se, have numerous alterations in their own metabolism, e.g., regarding fatty acid oxidation, redox status, and low grade inflammation [13]. Such metabolic alterations may impact oocyte metabolic status, placental structure and function, mammary gland development, and, in turn, milk composition. Since placental transport and breast milk are the only channels through which nutrients are transferred from mother to offspring, such alterations can affect the metabolic and endocrine status of the fetus, its early growth and neurological development and then the infant development. Concerning the breast milk, its components were shown to influence infant immune sensitization, microbiome, eating habits, nutrient utilization, and fat deposition beside breastfeeding benefits on maternal weight loss and maternal-newborn bonding [14,15]. Yet the impact of obesity-related alterations of breast milk on infant growth and health is incompletely described and the mechanisms underlying this multitude of effects poorly understood. Animal studies have begun to examine the mechanisms of fetal programming, with evidence that maternal obesity and the use of western high-fat high-simple sugar diet (WD) affect the differentiation and the function of the liver and the mammary glands during lactation, reduce milk yield, and modify its composition [16]. On fetus side, the adipose tissue is also impacted by an increase of adipogenesis combined to a modification of neural pathways of the hypothalamus promoting appetite [17]. The western diet, throughout pregnancy and lactation, could contribute to the increased, in breast milk of rodent models, of the leptin concentration, to the higher total fat and n-6 long-chain fatty acid (like arachidonic acid, ARA) content alongside the reduced level of n-3 long-chain fatty acid (like eicosapentaenoic (EPA) and docohexaenoic (DHA) acid) [14,16,18] although lipogenesis of the mammary gland is reduced [18]. This effect of the WD on breast milk composition was associated to a higher body weight of suckled pups [19]. The meta-analysis conducted by Ribaroff et al. [20] on 171 experimental studies identified that the exposure to high fat diet during the lactation period was a key modulator of bodyweight of the offspring at weaning and in adulthood, with a significantly greater effect on offspring fat mass than when WD exposure occurred during pregnancy alone [21]. High exposure to a WD during the breastfeeding phase is also associated with hyperinsulinemia, hyperglycemia, pancreatic islet hypertrophy, insulin resistance, hyperphagia, and overweight in male offspring [22]. Indeed, the development of dys-metabolic and non-alcoholic hepatic steatosis phenotype observed in offspring exposed to maternal obesity in the postpartum period has been suggested to be induced by factors in breast milk which probably modify the brain centers of energetic homeostasis [21]. These findings show that lactation is a vulnerable period during which transient insults can have lasting effects leading to impaired health outcomes in adulthood or accentuate the consequences of deleterious in utero environment, but with little knowledge to date on molecular actors. Additionally, maternal exposure to a high fat diet was associated with disrupted metabolism in offspring but with marked heterogeneity between studies, due to many confounding factors not taken into account such as dietary constituents, animal species, strain or gestational maternal weight gain [20]. Specific weaknesses in the experimental design predispose many results to biases that can contrast with human data. Indeed, the reported health benefits of breastfeeding, both in infancy and later in life, against the development of overweight and obesity (at least in childhood), hypertension, hyper-cholesterolaemia, type 2 diabetes, and non-alcoholic fatty liver disease [23] lead to conceiving the lactation period as an intervention window to compensate for certain deficits induced by fetal challenges. To better understand the putative beneficial role that breastfeeding could play in the face of the pandemic of obesity and the mechanisms underlying it, it is essential to first decipher the molecular descriptors of breast milk impacted by obesogenic perinatal challenge and to understand the extent to which they are associated with offspring outcomes in programming models.

The major aim of the present work, conducted in a rodent model, was to assess the impact of perinatal maternal moderate high caloric challenge on breast milk composition in relation with offspring metabolic development. To answer this question, (i) we assessed breast milk lipidome combined to free amino acids profiles in dams fed with WD during gestation and lactation; (ii) we evaluated the impact of milk lipidome and amino acids pattern of WD-fed dams on offspring growth and development; and (iii) we identified a set of molecular descriptors in breast milk that could be associated with blood lipidomic signatures in the offspring with a sex-specific manner. Our holistic approach, which incorporates data from several biological compartments (i.e., macro-nutriments, lipidome, and free amino acids in breast milk and lipidome in offspring blood) would improve our understanding of the molecular mechanisms linking the composition of breast milk with early growth of offspring in a high energetic perinatal environment. In particular, our findings highlight that (i) perinatal moderate high caloric diet impacts mature milk lipidome, providing important mediators that have the potential to metabolically program the offspring, such as high levels of sphingolipids and triglycerides-with unsaturated long-chain, that can be considered as main switches in the expression of enzymes involved in metabolism, thermo-regulation, and growth and (ii) pups nursed by moderate high caloric diet-fed dams have a specific blood lipidome with a sexual-dimorphism in the fatty acids-distribution of lipid species that could be associated to the differences observed between males and females in metabolic parameters and feeding behavior at weaning.

## 2. Results

### 2.1. Maternal Parameters during Gestation and Lactation Periods

**Figure 1:**
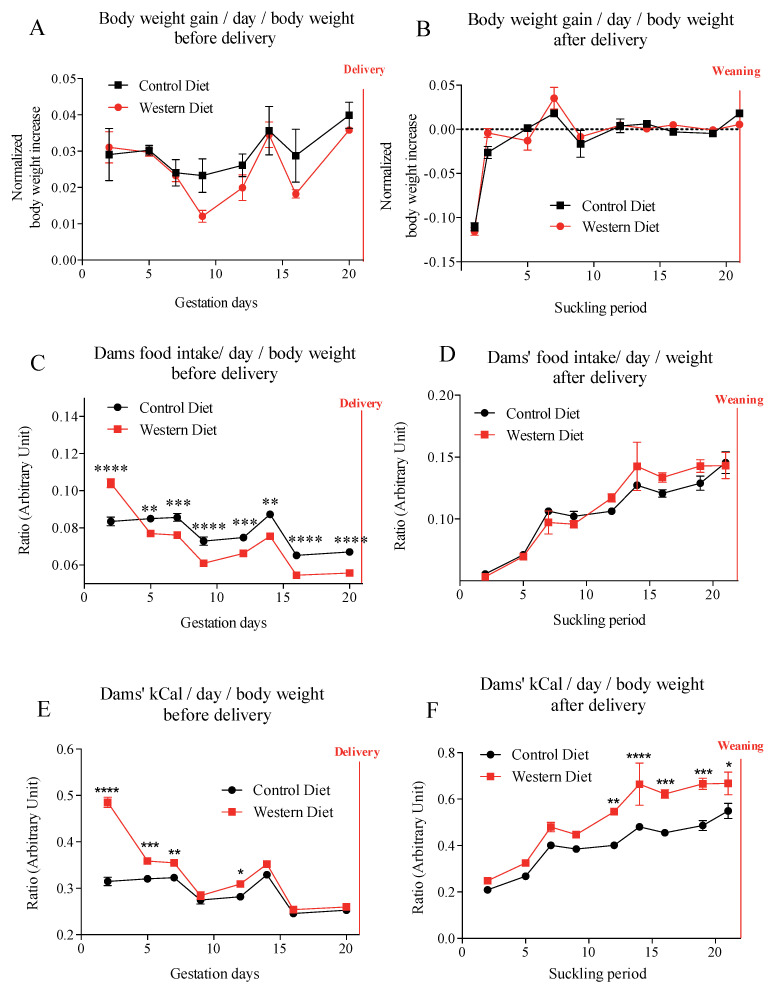
Dams’ body weight gain (**A**,**B**), food intake (**C**,**D**) and dams’ kcal intake (**E**,**F**) during gestation and lactation. Values of *p*-values (assessed by Mann–Whitney U test) between “western diet” and “control diet” groups were reported with *, **, ***, ****, significantly different; *p* < 0.05, *p* < 0.01, *p* < 0.001, or *p* < 0.0001, respectively.

Maternal body weight and body weight gain, in the control diet (CD) and western diet (WD) groups, were similar throughout both gestation and lactation periods (Figure 1A,B). During gestation period, females exposed to western-diet food ate more during the first 2–3 days than females exposed to the standard diet. After 3–4 days, WD-fed dams regulated their intake, with lower food consumption in quantity than control-fed dams (Figure 1C). Therefore, from day 8 these WD fed-dams absorbed the same amount of calories as the control females (Figure 1E). During lactation period, energy intake was higher for the WD-fed dams group from D12 of lactation compared to CD-dams and that, despite similar food intake (Figure 1D,F).

### 2.2. Pups’ Development during the Suckling Period

**Figure 2:**
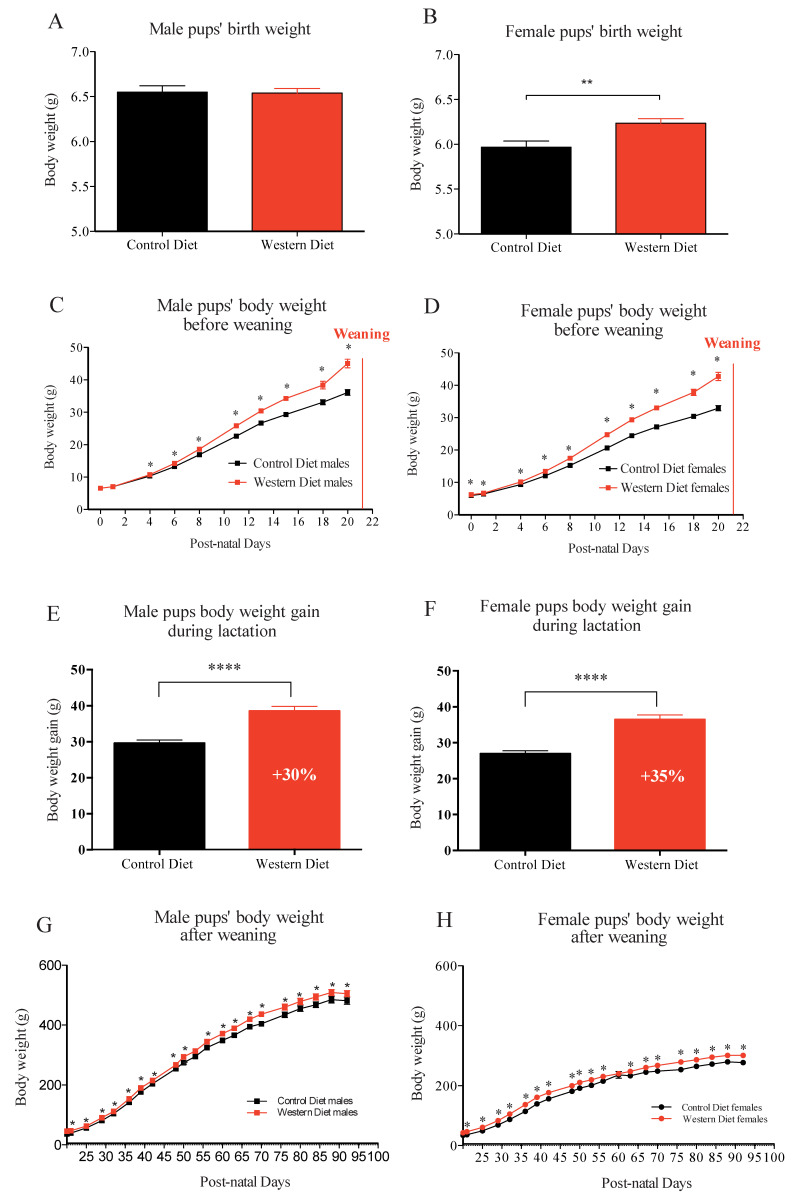
Male and female pups’ birth weight (**A**,**B**), Male and female pups’ body weight kinetic before weaning (**C**,**D**), the total body weight gain during lactation (**E**,**F**), and male (Paradis et al., 2017) and female pups’ body weight kinetic after weaning (**G**,**H**). Values of *p*-values (assessed by Mann–Whitney U test) between “western diet” and “control diet” groups were reported with *, **, ****, significantly different; *p* < 0.05, *p* < 0.01, or *p* < 0.0001, respectively.

Globally, male pups had birth weight higher than female pups, independently of dams’ diet. Pups’ body weights were similar in the two groups, at birth, in male (Figure 2A), (CD: 6.55 ± 0.07 vs. WD: 6.54 ± 0.05 with *p* = 0.92) but higher in female pups (Figure 2B), born from WD-fed dams, compared to those, born from CD-fed dams (CD: 5.97 ± 0.07 vs. WD: 6.24 ± 0.05 with *p* < 0.01). During the suckling period, pups’ weight gain was greater in WD group, with slight differences in sex, i.e., from birth to weaning, for female, and from D 5 of life until weaning, for male (Figure 2C,D). The total body weight gain during lactation was 30% higher for male pups born and fed from WD-fed dams than male pups from CD-fed dams (Figure 2E). Concerning the female, the total body weight gain during lactation was 35% higher for female pups born and fed from WD-fed dams than female pups from CD-fed dams (Figure 2F). From weaning to the end of the experiment (P95) under standard chow diet, higher body weight was maintained in the male and female offspring from WD dams compared to than from CD dams (Figure 2G,H).

### 2.3. Milk Macro-Nutriments Composition at Weaning

**Figure 3:**
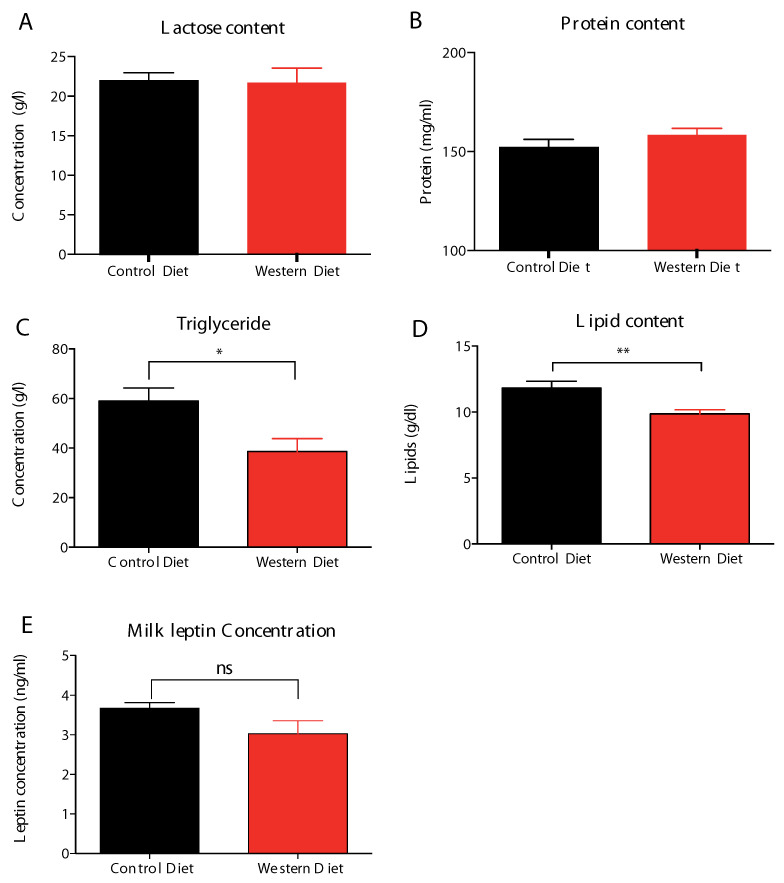
WD-derived mature milk content in lactose (**A**), protein (**B**), triglycerides (**C**), lipid (**D**), and leptin (**E**) at day 20 of lactation (L20). Milk total lipid (i.e., triglycerides and phospholipids) content (Figure 3**D**) was assessed as the sum of total fatty acid milk content. Values of *p*-values (assessed by Mann–Whitney U test) between “western diet” and “control diet” groups were reported with *, **, significantly different; *p* < 0.05, *p* < 0.01, respectively and “ns” for no significance.

Milk protein and carbohydrate contents were un-impacted (Figure 3A,B), in addition with similar milk leptin content (Figure 3E), whereas, total fatty acids (Figure 3D) and triglycerides (Figure 3C) were lower in milk provided by WD-dams, at the end of lactation.

### 2.4. Milk Free Amino-Acids Contents at Weaning

Dam milk free amino acids contents were very similar in both group, this was particularly the case of insulinemic and glycemic amino acids. WD-derived breast milk presented a trend to decreased levels in glycine along with a trend to higher levels in proline, citrulline and sulfur amino acids (Table 1).

### 2.5. Milk Total Fatty Acids Contents at Weaning

**Figure 4:**
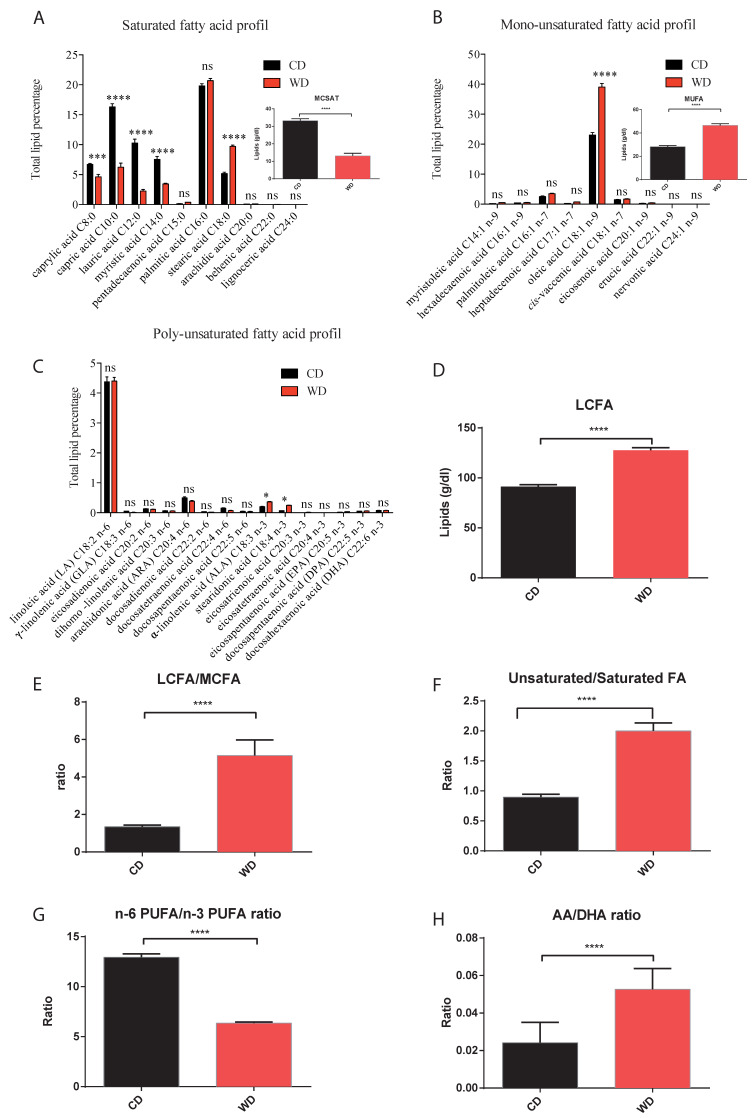
Milk profiles of total saturated (**A**), mono-unsaturated (**B**) and poly-unsaturated (**C**) fatty acids with total content in medium chain saturated and in mono-unsaturated fatty acids presented in miniature (**A**,**B**). Total content of long chain fatty acids (**D**) and long-chain/medium-chain fatty acids (**E**), unsaturated/saturated fatty acids (**F**), n-6 PUFA/n-3 PUFA (**G**) and AA/DHA (**H**) ratios at day 20 of lactation. AA: Arachidonic acid; DHA: Docosahexaenoic acid; MCSAT: Medium chain saturated fatty acids (C8:0 to C12:0); MUFA: monounsaturated fatty acid; LCFA: Long-chain fatty acid that contains at least 16 carbons; MCFA: Medium-chain fatty acid that contains less than 16 carbons. Values of *p*-values (assessed by Mann–Whitney U test) between “western diet” and “control diet” groups were reported with *, ***, or ****, significantly different; *p* < 0.05, *p* < 0.001 or *p* < 0.0001, respectively and “ns” for no significance.

In WD-fed dams, not only did the total milk fat content decrease, but so did the proportion of total medium-chain (C8- to C14-) fatty acids, and specifically medium-chain saturated fatty acids (Figure 4A), whereas total long-chain fatty acids (Figure 4D) proportion and the long-chain/medium-chain fatty acids ratio (Figure 4E) were enhanced, essentially due to higher stearic (Figure 4A), oleic (Figure 4B) and alpha-linolenic acids (Figure 4C). This WD-breast milk fatty acids profile was also characterized by a lower percentage in n-6 long-chain PUFA and a decreased n-6/n-3 total PUFA ratio (Figure 4G), but an enhanced AA/DHA ratio (Figure 4H), associated to a higher unsaturated/saturated fatty acids ratio (Figure 4F), essentially due to the higher content of the major mono-unsaturated oleic acid(C18:1).

### 2.6. Maternal Western Diet throughout Gestation and Lactation Discriminated Breast Milk Lipidome at Weaning

**Figure 5:**
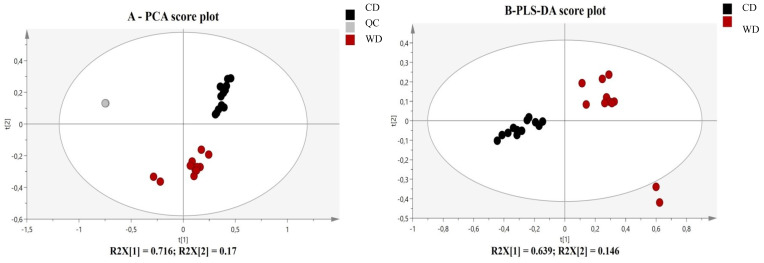

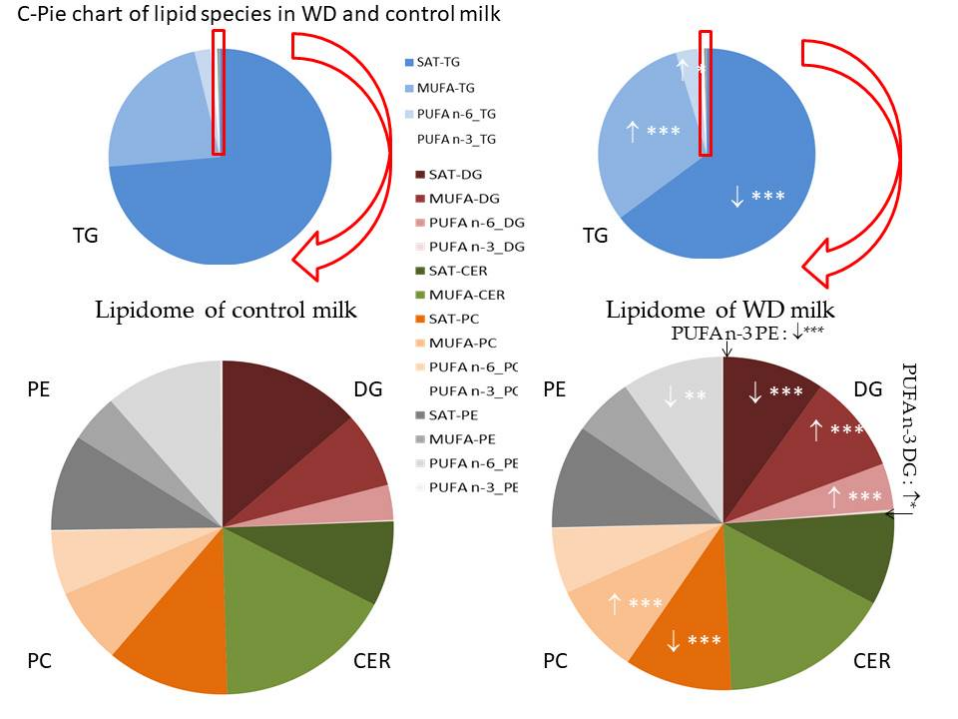
(**A**) PCA analysis reveals an influence of maternal high caloric diet during gestation and lactation on milk lipidome. PCA score plot on the two first dimensions of the PCA, accounting for 89% of the total variance for 3955 data points (features *m/z* acquired in LC- ESI^+^-HRMS), reveals natural clusters for milk provided by WD-(red) and C-(black) fed dams or QC samples (grey) at day 20 of lactation. (**B**) Score plots from PLS-DA classification between WD- and C-milk. (**C**) Pie chart showing the composition of the lipidome of breast milk from WD- and C-dams. The red box has been enlarged to visualize the distribution of diglycerides and phospholipids that are minors compared to triglycerides in breast milk. Values of p-values (assessed by Mann–Whitney U test) between “western diet” and “control diet” groups were reported with *, ** or ***, significantly different; *p* < 0.05, *p* < 0.01 or *p* < 0.0001, respectively. Concentrations (expressed in µM) of lipids ↑ increased and ↓ decreased in WD milk *versus* control milk.

Untargeted dam milk lipidomics phenotyping, using LC-HR-MS analysis, provided high throughput lipid species information on fatty acids distribution in various lipids families present in dam milk at the end of lactation. In the Principal Component Analysis (PCA) model validated by the high clustering of the QC, the lipidomic signatures showed a clear separation between milk lipidotypes provided by WD- and Control-fed dams (Figure 5A). Then, variables of importance in the projection (VIP) were selected in the supervised Partial Least Square Discriminant Analysis (PLS-DA) model applied on lipidomics profiles (Figure 5B), based on 1.5 as a cutoff-value, and annotated, using on line and in-house data base, and finally, quantified, using internal standards representatives of each lipids families in targeted analysis, as reported in Table 2. Although, milk provided by WD-fed dams presented similar median levels in total phospholipids and tri-and diglycerides families, we observed many significant discrepancies in several sub-groups of phospholipids (PLs), tri-and diglycerides (Figure 5C), likely due to significant differences in distribution of specific fatty acids in these families, in acyl chain length or in degrees of unsaturation (Table 2). Indeed, WD-milk presented (i) lower content in phosphatidylcholines (PC), diglycerides (DG) and triglycerides (TG) containing saturated fatty acids; (ii) higher content in PC, DG and TG containing MUFA; (iii) lower content in n-3 PUFA and n-6 PUFA-rich PE but higher levels in n-3 PUFA-rich DG and n-6 PUFA-rich DG and TG; (iv) lower content in short- or medium-acyl chain of PC and PE species(data not shown)and (v) higher levels in TG species with long acyl chain-length (i.e., TG (54:2)). Globally, this specific WD-breast milk lipidotype was associated to an enhanced unsaturated/saturated tails incorporated into the TG, DG and PC species, essentially with the contribution of oleic and linolenic acids.

Concerning the ceramides and sphingomyelins that discriminated both dam’s milk lipidotypes according to perinatal maternal diet, none contained PUFA either n-6 or n-3, on the other hand, sphingolipids containing medium-chain saturated fatty acids presented decreased levels whereas those containing stearic or oleic acids had enhanced levels in WD-milk lipidotype. Finally, among variables of interest to discriminate both milk lipidotypes, WD-milk provided similar total amounts of choline-PLs (i.e., PC, Cer and SM) (Table 2).

### 2.7. Maternal Western Diet throughout Gestation and Lactation Discriminated Offspring Blood Lipidome at Weaning with a Sexual Dimorphism Response

**Figure 6:**
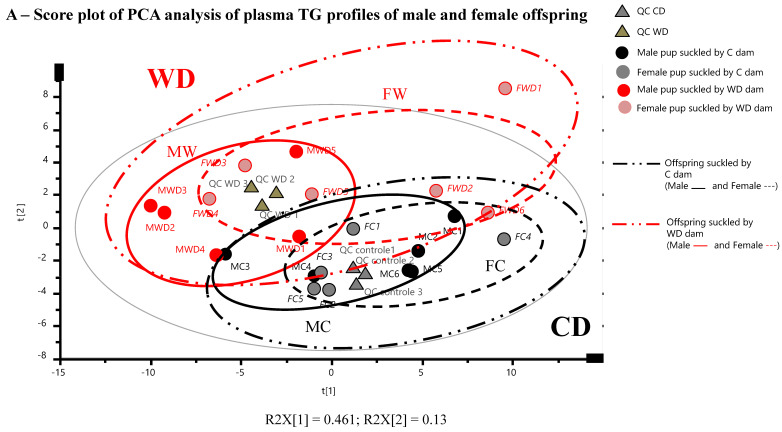

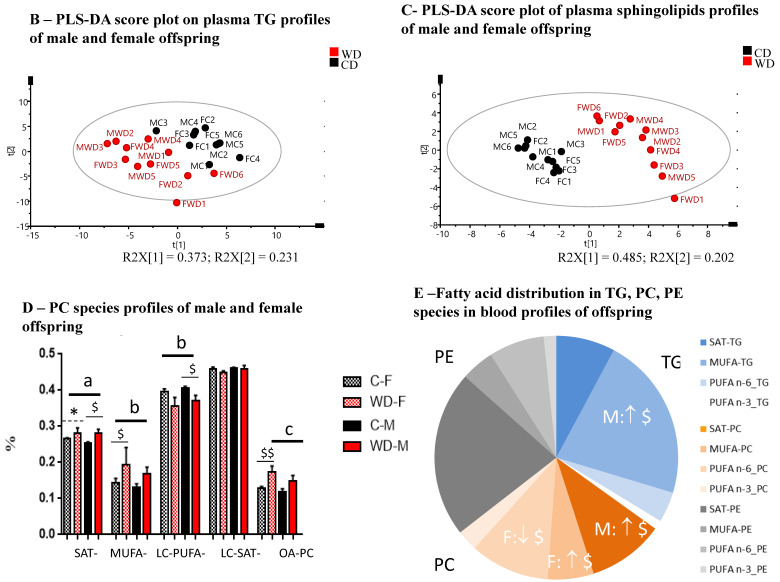
(**A**) PCA analysis reveals an influence of maternal diet during gestation and lactation on male and female offspring blood lipidome at day 25 of lactation period. PCA score plot on the two first dimensions of the PCA, accounting for 59% of the total variance for 62 data points (TGs), reveals natural clusters for all the male (MC, MWD) and female (FC, FWD) offspring and according to maternal diet (C, WD). The PCA model presented a cumulative modeled variation (R2X) value of 95% and 92% and a cumulative predicted variation (Q2Y) value of 83% and 14% for maternal diet and offspring sex groups, respectively, for 22 data points (Cer-SM), indicating good agreement. Score plots from PLS-DA classification of offspring blood TGs (**B**) and sphingolipids (**C**) species according to maternal diet (C, WD). Only one sample (FWD1) was identified as outlier according to the Hotelling’s T2 grey circle, corresponding to a multivariate generalization of the 95% confidence interval. (**D**,**E**) Fatty acids distribution in phosphatidylcholines (PC species), phosphoethanolamines (PE species) and triglycerides (TG species) in blood of male and female offspring of both WD and C groups (data of sphingolipids were detailed in Table 3). Values were mean ± SEM (**D**) or mean (**E**). For each biomarker, values of *p*-values (assessed by Mann–Whitney U test) between “western diet” and “control diet” groups, *regardless of sex group*, were reported with a, b, c significantly different; *p* < 0.05, *p* < 0.01 or *p* < 0.001, respectively. Values of *p*-values (assessed by Mann–Whitney U test) between “female” and “male” groups *considering each diet group*, were reported with *, *p* < 0.05 significantly different. Values of *p*-values (assessed by Mann–Whitney U test) between “western diet” and “control diet” groups *considering each sex group* were reported with $, *p* < 0.05 and $$ *p* < 0.01 significantly different. SAT: saturated fatty acids (C8:0 to C14:0); MUFA: Monounsaturated fatty acid; LC-SAT: Long-chain saturated fatty acids (that contains at least 16 carbons); LC-PUFA: Long-chain PUFA (polyunsaturated fatty acid that contains at least 16 carbons).

Unsupervised principal component analysis (PCA) carried out on offspring blood TGs (Figure 6A) or Cer-SM (data not shown) data obtained at weaning were validated by the high clustering of the QCs and allowed to identify both maternal diet groups (WD and C) associated to a sexual dimorphism, with slight overlap. Then, to address our classification purposes, we used a supervised partial least square–discriminant analysis (PLS-DA) on the same data sets to accurately model the relationships between groups. The PLS-DA model (Figure 6B) built with TGs data sets with a good predictive performance (R2 = 0.91 and Q2 = 0.69), internally validated by CV-ANOVA (*p*-value = 0.0015), revealed that each class (WD and C) was well separated, suggesting that the PLS-DA model successfully discriminated the blood TGs profiles of offspring suckled by WD-fed dams from those suckled by C-fed dams. When offspring sex was used as a discriminant parameter (data not shown) for the same data set (TGs), the model exhibited lower predictive performance. Considering offspring blood Cer-SM data set, the PLS-DA model (Figure 6C) presented good validation parameters (R2 = 0.95, Q2 = 0.83, CV-ANOVA *p*-value = 7.29 × 10^−8^) and exhibited also a very clear separation of WD and CD-pups groups. A similar clear clustering of offspring blood Cer-SMs profiles were obtained when considering sex as discriminant parameter (Supplementary Figure S1).

### 2.8. Maternal High Fat Diet Impacts Fatty Acids Distribution in Phosphatidylcholine, Sphingomyelin, and Triglyceride Species in Offspring Blood with a Sex-Specific Manner

In order to get a better evaluation of the effect of maternal perinatal diet group and offspring sex, and interactions between them, we carried out differential analyses on each sets of metabolites i.e., Cer-SM (Table 3) and TGs (Table S1) using 2-ways ANOVA. We found substantial differences in the offspring sphingomyelin profiles according to the maternal diet: the very-long chain Cer 24:0 and its associated ganglioside, the Lactosyl-Ceramide LacCer C24:0, were significantly increased in offspring suckled by WD-fed dams, specifically in females, whereas all species of sphingomyelin and especially, long-chain SM (SM 20:1 and SM 24:1) were decreased even more in males than in females offspring nursed with high fat diet-fed dams. Blood TGs profiles presented also lower proportions of long chain TG-containing saturated palmitic or stearic acids (TG (50:1) (C16:1_C18:0_C16:0) and TG(50:2) (C16:1_C18:0_C16:1)), and higher unsaturated long chain TG-rich in MUFA such as oleic acid (TG(54:5) (C18:1_C18:3_C18:1), TG(54:3) (C18:1_C18:1_C18:1), TG(53:2) (C15:0_C18:1_C20:1), TG(51:2) (C18:1_C15:0_C18:1) essentially in WD male group, compared to the corresponding C group. The proportion of PC-containing saturated and mono-unsaturated (oleic acid) were significantly increased respectively in male and female offspring suckled by WD-fed dams, along with lower PC-containing long chain PUFA and essentially PUFA n-6, in females (Figure 6D, E), and lower levels of PC with short and medium acyl chain length (data not shown), regardless of sex group. Fatty acid distribution in PE species was similar in WD and C-groups, independently of sex group. Finally, being fed with WD-derived breast milk had no impact on the global unsaturated/saturated tails incorporated into TG (Table S1) or PLs species (unpublished data).

## 3. Discussion

**Figure 7:**
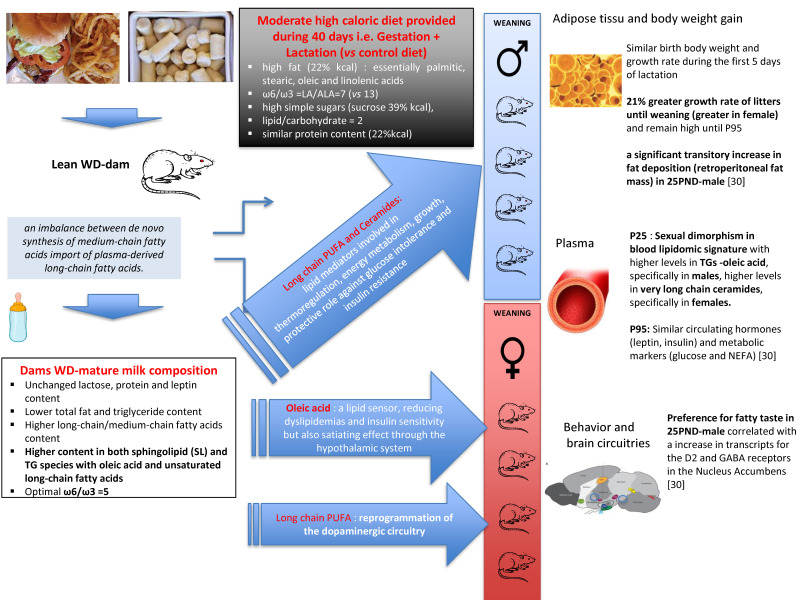
Putative mechanisms mediating the effects of moderate high caloric diet during gestation and lactation on mature breast milk composition and offspring blood lipidome and phenotype at weaning and at adulthood.

Lactation is a physiological state which presents significant challenges to maternal energy homeostasis due to its high need of energy. Indeed, the nutrients that a mother will consume during that period will have an impact on these challenges. In rodent experimentation, perinatal exposure to high energy diet is expected to impact mammary gland metabolism and milk composition, leading to specific changes in fuel utilization in the suckling pups when compared to perinatal exposure to a control diet. In the present study, we used an integrated metabolomics-lipidomics spectrometric platform for a wide-molecular profiling coupled to multidimensional statistical models to investigate breast milk phenotype in response to maternal high caloric-exposure during the perinatal period. Because diet is only consumed during gestation and lactation it does not induce obesity in the rat dams and allow us to link the breast milk phenotype and offspring metabolic outcomes to the moderate high caloric maternal diet *per se* and not to the maternal obesity status. Our results, obtained by this unique approach, reveal the effect of a high chronic energy intake throughout pregnancy and lactation on (i) the free amino acid pattern, the fatty acid profile and the phospholipid, sphingomyelin, and tri- and di-glyceride species of mature breast milk and on (ii) the blood lipidome of the weaned pups, that differ between groups in a sex-specific manner as summarized in Figure 7.

### 3.1. High Energy Exposure throughout Pregnancy and Lactation Impacts Specifically Breast Milk Metabo- and Lipidotype 

In the present study, dams were fed, during gestation and lactation only, a high caloric diet (WD) provided with high fat (22% kcal), high simple sugar (39% kcal), and 22% kcal protein content. Although chronic perinatal high energy feeding did increase the amount of dietary fat trafficked to the milk, the WD-fed dams provided breast milk with decreased total fat content whereas total amounts of protein, lactose, and leptin were preserved to the end of the lactation period. The preserved protein and carbohydrate levels in WD-dam breast milk is consistent with previous experimental paradigms [16,19,24] conducted on lactating dams fed diet with various calories content from fat (20–48%), protein (20–32%), and carbohydrate (17–62%) and showing that changes in diet, including the energy content, have very limited effects on protein and carbohydrate contents. On the other hand, the discrepancies between literature and our own data on low total fat and triglyceride found in breast milk of WD fed dams could be explained by the fact that above 2/3 of milk fatty acids found in breast milk arise from maternal body fat stores and less than 1/3 from maternal dietary intake [25]. Indeed, in above reported studies, increased breast milk fat content was probably the consequence of higher body fat stores in response to a longer period of high energy diet-exposure that started before mating and lasted until the end of lactation [19] or from weaning of dams [16], or to greater than 26–28% (kcal) of fat intake during gestation and lactation [16,24]. These conditions drastically differ with ours since in our study the dams were not obese, received the WD-diet for 40 days only and the diet contained only 22% (kcal) of fat. Interestingly in a model of C57/B6J obesity-prone mice, Wahlig et al. [25] investigated the effects of a high energy diet per se (presenting similar kcal carbohydrate (34%) and higher kcal fat (46%) to those used in our experimental paradigm) on metabolism of lactating obese or lean dams. These authors found that high energy diet, before and throughout gestation and lactation, reduced milk fat production by day 10 of lactation, in a significant way, only in obese dams without change in the protein or lactose content of the milk. These findings, combined to the important fall reported in breast milk fat content over day 15 of lactation in high energy diet-fed dams [19] argued in favor of the lower breast milk fat content observed in our WD-fed dams at day 20 of lactation, especially since dams lipid intakes in our experimental paradigm were twice lower than in Wahlig’s study [25]. Another interesting observation which is, once, again, to be related to the state of obesity in the lactating dams, and which is observed in the study by Bautista [16], is the higher level of leptin in breast milk at day 20 of lactation, which was not observed in our study. Associated to this low breast milk total fat content, WD induced lower breast milk triglyceride content and medium-chain saturated fatty acid percentage, whereas total long-chain fatty acid proportion (essentially oleic acid) as well as the long-chain/medium-chain fatty acids ratio were enhanced compared to control milk. By using tracer approach, Wahlig’s work [25] highlighted the fact that high fat-fed lean mice utilized more dietary fat for milk lipid synthesis relative to de novo lipogenesis in mammary gland. One of the direct consequences of this excess of traffic of dietary fat to the liver and the mammary gland is the decrease of both carbohydrate uptake and energy need for de novo synthesized milk lipid. This did not compromise milk production but simply adapted milk production to the available nutrients [25]. Indeed, the perinatal WD gave to lean dams has induced an imbalance between the de novo synthesis of medium-chain fatty acids in the mammary gland, using glucose or acetate as precursors, and the import of plasma-derived long-chain fatty acids, more probably released from TGs in chylomicra or very low density lipoprotein from liver than derived from the pool of non-esterified fatty acids in their adipose tissue. This imbalance could lead to an increase in both ratios of long- to medium-chain and unsaturated to saturated fatty acids in the WD-derived breast milk associated to a low total fat content, suggesting that the contribution of dietary fat (with higher content in long chain fatty acids, such as the saturated stearic acid and the unsaturated oleic and linolenic acids) to milk lipid composition did not compensate the decrease of de novo lipogenesis in the mammary gland. A similar higher ratio of saturated (palmitic acid), mono-unsaturated (oleic acid) and poly-unsaturated long-chain to total saturated medium-chain (C8:0–C14:0) fatty acids was found if we consider the distribution of fatty acids in the phospholipids, sphingolipids and triacylglycerides in the WD-derived breast milk. This enhanced long- to medium-chain fatty acids ratio in breast milk was already reported in other studies conducted with high long-chain fatty acids (as oleic acid) maternal intakes [15,18,26] but may be modulated also by the carbohydrate-to-lipid ratio of the diet [27]. In our experimental paradigm, control and western diet presented similar relative proportions of lipid and carbohydrate but enhanced simple sugars (glucose and dextrose), and saturated and unsaturated long-chain (essentially oleic and linolenic) fatty acids content in WD. Then, the enhanced long-chain fatty acid proportion along with the decreased triglycerides content measured in breast milk from WD-fed dams, reflected both dams fat intakes and the long chain fatty acids role as major regulators of de novo fatty acid synthesis in the lactating mammary gland [28,29]. The higher western diet-content in simple, rapidly oxidized sugar, is consistent with promotion of a greater milk production but also with mammary de novo lipogenesis (and the associated energy requirements), which has a significant contribution still occurring in WD-fed lean dams albeit likely to a lesser extent than in control diet-fed lean dams. Indeed, the higher milk production estimated in high fat-fed lean dams by Wahlig et al. [25], using milk energy output and milk composition measurements, may explain the sustained enhanced growth of pups observed in our present study. Indeed, it can be assumed that pups modify their suckling-time and therefore the total volume of milk consumed according to the energy intake of the milk. This can also be related to the observation that WD dams even though they consumed more calories during the lactation period did not gain more weight than CD dams.

### 3.2. Pups Suckled with WD-Derived Breast Milk Present Changes in Energy Fuel Utilization and Metabolism

Similarly to Nicholas et al. study [19], we reported a similar birth body weight and a similar growth rate of all litters for the first 5 days of lactation. On the other hand, the establishment of lactation was associated to a 21% greater growth rate of litters, thereafter and until weaning, for rats fed a high-energy diet, with a sex-specific manner (greater in female). We previously reported in the same experimental study [30] that, on offspring fed with standard chow diet from weaning to the end of the experiment (P95), body weight remained higher for the offspring from WD dams than from CD dams associated with similar circulating hormones (leptin, insulin) and metabolic markers (glucose and NEFA) until P95. Especially, offspring from WD-fed dams presented a significant transitory increase in fat deposition (retroperitoneal fat mass ratio) at P25 only. A possible explanation for these differences in offspring growth trajectory from 5 days of lactation may be due to the fact that pups, nursed by WD-fed dams, suckled low-energy breast milk with higher content in long-chain than medium-chain fatty acids, mostly incorporated in phospholipids and triacylglycerides. Although, medium-chain fatty acids are more directly available to the suckling rat, the longer-chain analogues have different metabolic effects [18] and whose distribution in phospholipids and/or triacylglycerides may optimize their utilization by the suckling offspring. For example, DHA supplementation was reported to be associated with a higher brain accretion when provided as a phospholipid than supplied as part of a triglyceride [31]. Moreover, besides being important structural elements of membranes, long-chain polyunsaturated fatty acids are firmly implicated in gene expression of nuclear transcription factors such as peroxisome proliferator activated receptors (PPARs) and sterol regulatory element binding proteins (SREBPs). The latter play key roles in the expression and/or repression of a variety of enzymes involved in intermediary metabolism, thermo-regulation, energy partitioning or in growth and differentiation [32]. Moreover, the fact that our 25 PND-male pups fed WD-derived milk with a high AA/DHA ratio and presented a transitory increase in fat-deposition can also be related to the fact that, in humans, a correlation is reported between the perinatal exposure to a high AA/DHA ratio during the first months of life and infant adiposity [15]. In addition, the potential functionality of the mono-unsaturated oleic acid, that was greatly enhanced in dam milk lipidome and offspring blood TG and PC species, remains to be explored in infant and its nutritional relevance needs to be further investigated [33]. Therefore, it cannot be excluded that the higher growth observed in pups suckled by WD-fed dams could be exacerbated by enhanced milk output as suggested above in the discussion. Interestingly, WD-fed dams provided specific breast milk with higher total content in stearic, oleic, and linolenic acids, enhanced levels in sphingolipid and TG species with long acyl chain length or with less than four degrees of unsaturation but lower levels of PC and TG species with short and medium acyl chain length. As reported in another study [34], this WD-metabotype milk was associated with an offspring blood lipidomic signature, in a sex-specific manner. Pups nursed by WD-fed dams presented higher levels in TGs rich in oleic acid, specifically in males, increased levels in very long chain ceramides, specifically in females, but decreased levels in TGs-containing saturated stearic acid, along with lower levels of PC species, regardless of acyl chain length, but unchanged unsaturated/saturated tails incorporated in TG and PLs species, regardless of sex group. Considering that (i) early exposure to n-3 PUFAs (such as linolenic acid) prevented the hepatic lipid accumulation, reduced glucose and non-esterified fatty acid serum levels in 21-day-old pups [35], (ii) long-chain sphingolipid species such as C24 ceramide exhibit a protective role against the development of glucose intolerance and hepatic insulin resistance [36,37], and (iii) oleic acid was identified as a lipid sensor, reducing inflammation and dyslipidemias as well as improving insulin sensitivity, likely through NAD-dependent deacetylase sirtuin-1 (SIRT1)- Peroxisome proliferator-activated receptor gamma coactivator 1-alpha (PGC-1α)-dependent fatty acid oxidation pathway [38], we suggested that maternal consumption of both PUFAs and oleic acid during pregnancy and lactation impacts breast milk lipid composition. Indeed, in our experimental paradigm, WD-fed lean dams provided milk with high content in oleic and linolenic acids and an optimal n-6/n-3 PUFA ratio around 5 that could counterbalance many harmful effects of a perinatal high calorie diet exposure and might favorably influence the lipid profile and the antioxidant status of the offspring at least during the lactation period. Additionally, because fatty acids act as signaling molecules and sensors of whole-body energy status, both human and rodent studies provide direct and indirect evidence for their central action after delivery via TG hydrolysis. Fatty acids are involved in a multitude of neural responses that can modify the hypothalamic control of energy homeostasis (e.g., nutrient storage and mobilization) [39], feeding [40] and modulate hedonic food intake in reward circuitry (e.g., dopamine signaling) [41,42]. Indeed, the direct infusion of oleic acid in the central nervous system highlighted the satiating power of this FA over the hypothalamic homeostatic system by slowing down food intake via the decreased expression of neuropeptide Y [43] or inhibition of carnitine palmitoyltransferase-1 (CPT1)-dependent fat oxidation [43,44]. Moreover, according to Berland et al. [45,46], local accumulation of free fatty acids in the dopaminergic mesolimbic system, following dietary TG-hydrolysis, can directly target the mesolimbic system to regulate amphetamine-induced locomotion and to release dopamine, reinforcing motivational aspects and reward seeking behavior likely by a control of D2 receptor signaling. The authors suggested that one of the cellular mechanisms of TG action in the mesolimbic system can be upstream of dopamine 2 (D2)-receptor. The fact that our 25 PND-male pups suckled by WD-fed dams presented, compared to pups suckled by control dams, a significant increase in both fat deposition (retroperitoneal fat mass ratio) and preference for fatty taste ([30] for males and data not shown for females) that is correlated with an increase in transcripts for the D2 and GABA receptors in the Nucleus Accumbens could be in agreement with this hypothesis [30]. Moreover, the sexual dimorphism observed in the blood lipidome of our male and female pups nursed by WD-fed dams could be associated to the differences we observed between males and females in metabolic parameters and feeding behavior [30] at weaning. Additionally, this WD-derived lipidome of weaned pups may also be consistent with the reprogrammation of the dopaminergic circuitry very recently reported in offspring suckled by mice fed WD only during lactation and the sexually dimorphic expression of dopamine-related phenotypes, i.e., hyperlocomotion in males and increased intake of palatable food and sucrose in females, but at 6 months of age [47].

To conclude, feeding a high caloric diet, including a two-fold carbohydrate-fat ratio with a high proportion of oleic acid, n-3 PUFA and single sugars during both gestation and lactation induces an adaptation of dams’ energy intake during gestation, a higher caloric intake during lactation without obesity development. This high caloric challenge during the perinatal period was associated with change in breast milk composition, thereby providing important mediators that have the potential to metabolically program the offspring and impact the developmental trajectory of organs such as brain and liver. However, a limitation of the present study is that to decipher the information which has passed through the milk, a cross-fostering is necessary, in order to avoid the prenatal effects of a high-caloric exposure that can induce a higher birth weight. However, a recent meta-regression analysis identified high fat diet (HFD) exposure during the lactation period (but not the gestational HFD) as the key modulator of body weight, adiposity, triglyceridaemia, cholesterolaemia, and insulinaemia in both female and male offspring [20]. Despite this bias, the profiles of free amino acid and fatty acid that we identified in breast milk after exposition to WD reflect an early adaptation of the metabolism of the mammary gland to manage high energy-exposure and the resulting oxidative stress, due to the fact that mammogenesis and lactogenesis occurred early from the mid-pregnancy [28,48]. This WD-derived breast milk provides molecular biomarkers that represent energy sources, but also sensors of cellular energy status and regulators of cell metabolism and physiology, mostly being involved in metabolic, neurological, and gastrointestinal neonatal development. Milk volume is yet another important variable that determines pups’ energy intake and thus impact their outcome. Therefore, it cannot be excluded that WD-fed dams, although not obese, provided less milk output. In line with the Developmental Origins of Health and Disease (DOHaD) concept and the lactation programming hypothesis, WD-derived breast milk exposure impacts offspring blood lipidomic phenotype at weaning, with a specific sexual-dimorphism in the fatty acid distribution in phospholipid, triglyceride and sphingolipid species that could have functional effects on offspring metabolism by altering target genes and, in turn, influence its phenotype and its behavior.

## 4. Material and Methods

### 4.1. Ethics Statement

All animal procedures were carried out in accordance with current institutional guidelines on animal experimentation in the EU (directive 2010/63/EU) and in France (French Veterinary Department—A44276).The experimental protocol was approved by the Animal Ethics Committee of Pays de La Loire and registered under reference CEEA.2012.208 (Angers, France) (10 January 2013) with a particular attention to minimize stress and the number of animals used in each series of experiments in compliance with the commonly-accepted ‘3Rs.

### 4.2. Animals and Diets

**Figure 8:**
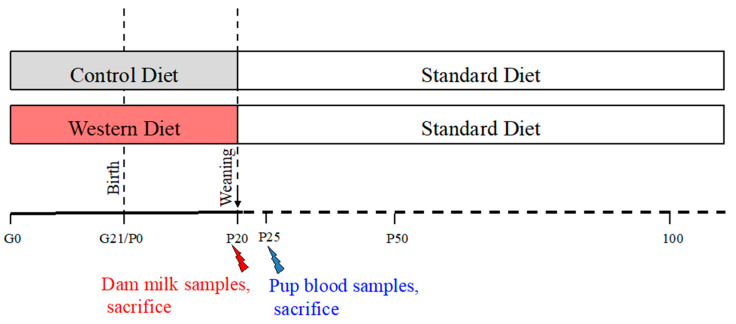
Schematic diagram of the study design. Sixteen female Sprague–Dawley (SPD) rats at gestation day 1 were fed either a control diet for 8 of them or a western diet for the others during gestation and lactation period. Red spark: At day 20, dam milks were sampled and dams were then sacrificed. The offspring were all kept onto standard chow diet until the end of the experiment (P100). Blue spark: At P25 a group of 6 males and 6 females were sacrificed.

Animal were housed in climate-controlled room with constant air humidity, a temperature of 22 ± 2 °C, and a 12 h light/dark cycle and with food and water ad libitum. Sixteen female Sprague–Dawley rats (Janvier, Le Genest Saint Isle, France) were purchased at gestation day 1 (G1) with a mean bodyweight of 240–290 *g*. Pregnant dams were transferred into individual polycarbonate cages, and, from G1 through the end of gestation, were randomly assigned to receive, during the gestation and lactation periods, a control diet (CD) (5% beef fat and 0% sucrose), *n* = 8/group, or a western diet (WD) (21% beef fat, with higher content in palmitic, stearic, oleic and linolenic acids, and 30% sucrose), *n* = 8 (as previously described [30], Table 4). At birth, litter size was adjusted to eight pups per litter with a 1:1 male to female ratio. At weaning (P21), the offspring born to CD and WD dams were kept onto standard chow until the end of the experiment at P100 (Figure 8). At day 25, 6 males and 6 females were sacrificed and their blood was collected in tubes with EDTA (Laboratoires Léo SA, St Quentin en Yvelines, France) and centrifuged at 2500× *g* for 15 min at 4 °C. Plasma was frozen at −20 °C for further analysis.

### 4.3. Milk Sampling

At 20 days of lactation, CD and WD lactating dams were milked as described previously [49] and at the same time of the day to prevent the bias due to circadian variations in milk production. Briefly, pups were separated from their mothers 1 h and dams were milked manually under light anesthesia, through inhalation of 1.5 L/min of oxygen with 2% to 2.5% of isoflurane, and 20 min after an intraperitoneal injection of 1 unit of oxytocin (Syntocinon; Sigma-Tau, Ivry sur Seine, France) The milk collected (0.3 to 0.8 mL) from each dam was immediately frozen at −20 °C until subsequent analysis.

#### 4.3.1. Milk Leptin Analysis

Dam milk leptin content was assayed with specific ELISA kits following the manufacturer’s instructions (rat leptin ELISA kit, Linco Research, St. Charles, MO, USA).

#### 4.3.2. Targeted Free Amino Acid (FAA) and Fatty Acid (FA) Analysis

Dam milk total fatty acids (FAs) were analyzed by gas chromatography using an Agilent Technologies 7890A^®^ instrument (Perkin Elmer, Villebon-sur-Yvette, France), following milk extraction of lipophilic metabolites, using the modified liquid–liquid extraction method Bligh–Dyer [50], and trans-esterification [49].

Determination of free amino acid (FAA) concentrations in dam milk by Ultra Performance Liquid Chromatography-Mass Spectrometry in tandem (UPLC-MS/MS) was performed as previously described [51]. Briefly, the protocol comprised a delipidation step by centrifugation, followed by a deproteinization step by addition of sulfosalicylic acid and centrifugation. FAA from supernatant were finally derivatized using AccQ^®^TagTM Ultra reagent, separated on liquid chromatographic using an Acquity H-Class® UPLC system (Waters Corporation. Milford, MA, USA) combined to a Xevo TQD® mass spectrometer (Waters Corporation, Milford, MA, USA), then, identified and quantified, using the Waters TargetLinks^TM^ software (Waters Corporation, Milford, MA, USA).

#### 4.3.3. Liquid Chromatography-High-Resolution-Mass Spectrometry (LC-HRMS)–Based Lipidomic Profiling and Lipid Species Quantification

Dam milk untargeted lipidomics fingerprinting was performed as previously described [52]. One quality control (QC) sample was prepared by pooling 10 µL from each individual dam milk sample and repeatedly injected throughout the analytical batch for correction and normalization purposes. Briefly, following Bligh–Dyer extraction of dam milk and QC samples (50 µL), the organic layers were dried and subsequently reconstituted in acetonitrile-isopropanol-water (ACN:IPA:H2O 65:30:5, *v/v/v*). Ultra-Performance-Liquid Chromatography-High-Resolution Mass Spectrometry (UPLC-HRMS) analyses were performed on a 1200 infinity series^®^ HPLC system (Agilent Technologies, Santa Clara, CA, USA) coupled to an Exactive Orbitrap^®^ mass spectrometer (Thermo Fisher Scientific, Bremen, Germany) equipped with a heated electrospray (H-ESI II) source (operating in polarity switch mode). Samples were randomized and injected (5 µL) altogether with QC extracts onto a reversed-phased Charge Surface Hybrid (CSH) ^®^ C18 (2.1 mm × 100 mm. 1.7 μm) column (Waters Corporation, Milford, MA, MA), using ACN:H2O (60:40) and IPA:ACN:H2O (88:10:2) as solvent A and B, respectively; both containing 10 mM ammonium acetate and 0.1% acetic acid and with a flow-rate of 300 µL/min.

Quantification of lipid species in dam milk and offspring blood were performed using targeted UPLC-HR-MS/MS analysis. Quality control (QCs) samples were prepared by pooling 10 µL from each individual dam milk sample (milk QC sample) or 10 µL from each individual blood sample of both WD (WD blood QC) and control (C blood QC) groups. Lipids were extracted from dam milk, offspring blood and milk and WD and C blood QC samples (100 µL) as reported previously [52]. In brief, 225 µL of ice-cold methanol were initially added to defrost samples, then, 750 µL of ice-cold methyl-*ter*-butyl ether (MTBE) containing exogenous internal standards [10 µmol/L; Cer (d18:1/17:0), SM (d18:1/17:0), CE (19:0), *d*_62_-PC (32:0), *d*_62_-PE (32:0), and *d*_5_-TG (50:0); Avanti Polar Lipids, Inc., Alabaster, AL, USA] were added as well as 113 µL of water. The final mixture was centrifuged (10.000 *g*, 10 minutes, 5 °C) and 600 µL of supernatant were evaporated to dryness under a nitrogen stream (room temperature). Dried samples were reconstituted with 110 µL mixture of acetonitrile/isopropanol/water (65/30/5, *v/v/v*). Lipid species analysis was performed on a Synapt ^TM^ G2 HRMS Q-TOF mass spectrometer, equipped with an electrospray ionization (ESI) interface operating in the positive and negative ionization mode, combined to an Acquity H-Class® UPLC^TM^ device (Waters Corporation, Milford, MA, USA). Samples and QC extracts were randomized and injected (5 µL) on to an Acquity UPLC CSH C18 1.7 µm, 100 × 2.1 mm reverse-phase column (Waters Corp., Milford, MA, USA).

#### 4.3.4. Lipidomics Data Analysis and Lipid Species Characterization

Dam milk lipidomics fingerprinting generated LC–ESI^+/-^-HRMS raw data files that were preprocessed with Xcalibur 2.2^®^ (Thermo Fisher Scientific, San Jose, CA, USA). Then, Workflow4Metabolomics® (W4M) (http://workflow4metabolomics.org) was used for alignment, integration and extraction of the peak intensities following normalization of intra- and inter-batch effects by fitting local polynomial regression models to QC samples. The resulting detected [*m/z*; RT] features (i.e., ions of given mass-to-charge ratio and retention time) was then manually sorted out according to their quality of integration using a 30% relative SD cutoff within the repeated pooled QC injections [52]. This processing provided a list of [*m/z*; RT] features of interest for each sample. Thereafter, the global identification of lipids was performed by using the LIPID Metabolites and Pathways Strategy database (http://www.lipidmaps.org/) in which, the lipid exact mass measured (mass error less than 5 ppm) takes into account the specifically formed adduct and the structural elucidation of the detected lipids was performed according to their elemental composition as previously described [52].

Data acquisition of targeted lipid species analysis was achieved using MassLynx^TM^ software version 4.1 (Waters Corporation, Milford, MA, USA), for retention time alignment and for automatic integration (expressed as µM compared to *adhoc* internal standard response). The lipid species observed in dam milk or offspring blood were mainly triacylglycerol (TG), phosphatidylethanolamine (PE), phosphatidylcholine (PC), sphingomyelin (SM), ceramide (Cer) and diacylglycerol (DG). The major lipid species PC, PE, SM, and Cer were detected in ESI^+^ as protonated ions ([M + H]^+^); while ammonium adducts ([M + NH_4_]^+^) were preferentially formed for DG, TG. The negative mode provides information about the fatty acid chain length ([M − H]^−^). Moreover, MS data were simultaneously acquired in fast polarity switching mode operating in MS and MS/MS (All Ion Fragmentation) full-scan analyses at high mass resolution, helping us to identify the proposed lipids by examination of the (pseudo) tandem mass spectrometry spectrum generated, combined with the use of an in-house reference databank [53]. The annotation and the naming structure for all lipid classes were performed according to the LipidMAPS nomenclature.

### 4.4. Statistical Analyses

Data are expressed as mean ±/- SEM in tables and figures. Differences among nutritional groups (WD vs. CD) or between male and female groups, considering each diet group, were analyzed by the non-parametric Mann-Whitney U-test using GraphPad Prism^®^ software version 6.00 (La Joya. California, USA), due to the non-normality of the data and the small size of the groups (*n* = 12 observations per nutritional group or *n* = 5–6 observations per sex group). Dams’ food intake and Kcal intake were assessed using a Two Way ANOVA followed by a Dunn’s post-hoc test with dam’s diet and age factors. The kinetic of the pups’ body weight were assessed using a two way ANOVA followed by a Dunn’s post hoc test with dam’s diet and offspring sex factors, whereas targeted blood lipid species were analyzed with two-way ANOVA with dam’s diet and offspring sex factors, followed by Sidak’s multiple comparisons post-hoc test for comparisons between both diet groups for each sex. For all data analyses, the significance level (α) was set to 5%.

Untargeted lipidomic [*m/z*; RT] features were firstly column-wise centered and scaled with a Log-Pareto scaling and then, analyzed by multivariate statistical analysis, using SIMCA P^®^ version 13 (Umetrics AB. Sweden). Principal component analysis (PCA), which is an unsupervised method, was firstly performed on the lipidomic LC- ESI^+/-^-HRMS spectral data matrix to visualize the general structure of the dams’ milk lipidotypes and identify potential atypical or outlier observations. Subsequently, the susceptibility to perinatal maternal diet (western diet) was assessed by using the supervised Partial Least Squares Discriminant Analysis (PLS-DA) in order to pinpoint the descriptors, which best contribute to the discrimination of the two dams’ milk groups. The prediction ability of the PLS-DA models were fitted with good cumulative R2X (proportion of the total variance of the dependent variables that is explained by the model) and R2Y (fraction of y response-variation, i.e., the group membership variables, modeled in the component) and Q2 (overall cross-validated R2Y for the component).

## Figures and Tables

**Table 1:** Concentration levels of free amino acids in milk (µM) provided by dams fed Western vs. Control diet at D20 of lactation (L20).

Free Amino Acid Concentrations (µM)	Control Diet (*n* = 12)	Western Diet (*n* = 12)	Mann-Whitney Test *p*-Value
EAA	2248 ± 770.8	2595 ± 874.5	0.2624
Histidine	51.16 ± 29.05	61.03 ± 23.883	0.2537
Arginine	37.87 ± 22.38	44.43 ± 37.75	0.2104
Isoleucine	27.16 ± 13.68	26.91 ± 20.23	0.5426
Leucine	76.09 ± 35.84	63.69 ± 33.65	0.3724
Proline	401.65 ± 124.76	544.55 ± 169.73	0.0775
Methionine	130.62 ± 57.28	169.65 ± 64.76	0.1764
Lysine	529.72 ± 253.02	514.97 ± 247.18	0.7430
Phenylalanine	46.10 ± 20.42	37.84 ± 19.66	0.1957
Threonine	800.84 ± 269.045	969.85 ± 384.32	0.2876
Tryptophan	45.16 ± 9.45	49.12375 ± 18.45	0.4364
Valine	106.14 ± 37.38	113.13 ± 57.38	0.8729
NEAA	4078 ± 987.2	4275 ± 1187	0.6599
Alanine	1072.63 ± 292.15	1306.35 ± 483.61	0.1418
Aspartic acid & asparagine	501.03 ± 149.50	462.2875 ± 142.92	0.5806
Glutamine	89.29 ± 44.26	58.22 ± 23.27	0.0881
Glutamic acid	969.73 ± 237.99	969.11± 208.5	0.8729
Glx	1059 ± 264.4	1027 ± 225.2	0.9616
Glycine	445.41 ± 155.76	316.53 ± 112.61	0.0593
Serine	600.34 ± 191.48	651.07 ± 237.48	0.4037
Tyrosine	65.75 ± 31.99	57.62 ± 30.98	0.5806
Taurine	306.81 ± 78.03	341.79 ± 57.93	0.0881
Citrulline	72.57 ± 16.82	90.75 ± 39.68	0.0679
BCAA	209.4 ± 83.87	203.7 ± 110.7	0.6197
Insulinemic and glycemic amino acid	1578 ± 570.2	1733 ± 668.7	0.4704
SAA	449.5 ± 119.5	532.9 ± 111.8	0.0775

Values are mean ± SD. EAA: Essential amino acids; NEAA: Non-essential amino acids; FAA: Free amino acid; Glx: glutamine + glutamic acid; BCAA: branched chain amino-acids (Valine + Leucine + Isoleucine); Insulinemic and glycemic amino acids = valine + leucine + isoleucine + lysine + threonine + arginine. Sulfur Amino Acids (SAA): taurine + methionine.

**Table 2:** Concentrations of annotated lipids that discriminated (* VIP > 1.5 in PLS-DA model (components 1–2)) lipidotypes of breast milk provided by dams fed control or western diet at day 20 of lactation period.

			Concentrations	
Lipids	mz	Median (25% and 75% Percentile) at D21		Mann–Whitney U *p*-Value
		Control Diet (*n =* 8)	Western Diet (*n =* 8)	
Triacylglyceride (TG)		6.00 (1.68–3.43) mM	3.19 (2.03–5.42) mM	0.0887
TG (26:0) TG(8:0_8:0_10:0)	516.4272 [M + NH_4_]^+^	164.2 (127.4–300.3)	109.7 (55.12–171.2)	0.0288
TG (28:0) TG(10:0_10:0_8:0) *	544.4587 [M + NH_4_]^+^	474.2 (336.8–612.7)	289.5 (132.1–436.8)	0.0627
TG (30:0) TG(8:0/10:0/12:0) *	572.4902 [M + NH_4_]^+^	454.5 (283.1–591.2)	198.3 (131.6–318.6)	0.0068
TG (32:0) TG(12:0/10:0/10:0) and iso TG(8:0/10:0/12:0) *	600.5186 [M + NH_4_]^+^	438.0 (282.2–621.5)	179.6 (99.2–328.2)	0.0052
TG (34:0) TG(10:0_10:0_14:0) and iso TG(8:0_12:0_14:0) *	628.5530 [M + NH_4_]^+^	309.3 (195.3–476.4)	134.4 (72.3–222.8)	0.0052
TG (36:0) TG(10:0_12:0_14:0) *	656.5815 [M + NH_4_]^+^	209.1 (125.4–336.3)	89.89 (46.35–153.2)	0.0089
TG (36:1) TG(12:0_12:1_12:0)	654.5685 [M + NH_4_]^+^	300.9 (181.9–377.8)	217.1 (151.6–430.3)	0.9502
TG (38:0) TG(10:0_16:0_12:0) *	684.6127 [M + NH_4_]^+^	125.1 (77.48–214.1)	52.33 (26.24–93.34)	0.0147
TG(39:0) TG(8:0_16:0_15:0) *	698.6282 [M + NH_4_]^+^	11.80 (5.84–18.05)	10.73 (4.17–17.88)	0.8357
TG (40:0) TG (8:0_16:0_16:0) *	712.6456 [M + NH_4_]^+^	60.58 (34.59–99.80)	25.28 (13.70–52.79)	0.0288
TG (40:1) TG (8:0_16:0_16:1) *	710.6284 [M + NH_4_]^+^	246.2 (143.1–334.5)	126.0 (79.54–260.7)	0.0749
TG (40:2) TG (8:0_16:1_16:1) *	708.6143 [M + NH_4_]^+^	157.8 (90.57–191.0)	82.74 (47.88–149.0)	0.0887
TG (42:1) TG(10:0_14:0_18:1) *	738.6616 [M + NH_4_]^+^	241.6 (144.7–327.0)	125.9 (85.28–258.3)	0.0749
TG (42:2) TG (10:0_14:1_18:1) *	736.6440 [M + NH_4_]^+^	154.1 (92.78–199.0)	90.98 (68.16–179.6)	0.3867
TG (42:3) TG (10:0_14:1_18:2)	734.6301 [M + NH_4_]^+^	58.11 (34.04–73.95)	37.86 (19.68–67.31)	0.3104
TG (42:4) TG (10:0_14:1_18:3)	732.6140 [M + NH_4_]^+^	24.38 (14.90–37.17)	15.27 (7.63–25.41)	0.1419
TG (43:0) TG (14:0_15:0_14:0)	754.6937 [M + NH_4_]^+^	6.37 (5.86–8.58)	12.41 (6.04–14.34)	0.0437
TG (43:1) TG (14:0_15:0_14:1) *	752.6761 [M + NH_4_]^+^	32.20 (17.46–37.00)	29.89 (13.20–50.73)	0.5678
TG (43:2) TG (14:1_15:0_14:1) *	750.6604 [M + NH_4_]^+^	16.03 (8.92–18.99)	23.53 (10.74–41.53)	0.1044
TG (44:0) TG (10:0_16:0_18:0)	768.7029 [M + NH_4_]^+^	31.70 (18.15–42.22)	14.85 (9.33–30.34)	0.1220
TG (44:1) TG (10:0_16:0_18:1) *	766.6916 [M + NH_4_]^+^	205.8 (121.7–280.3)	100.9 (62.17–222.7)	0.1639
TG (44:2) TG (10:0_16:1_18:1) *	764.6769 [M + NH_4_]^+^	145.5 (84.44–179.1)	89.53 (53.26–174.3)	0.4285
TG (44:3) TG (10:0_16:1_18:2) *	762.6597 [M + NH_4_]^+^	90.97 (50.12–107.0)	62.81 (36.39–123.9)	0.8357
TG (44:4) TG (10:0_16:1_18:3)	760.6466 [M + NH_4_]^+^	42.93 (22.53–57.29)	28.94 (11.95–57.16)	0.6182
TG (45:0) TG (15:0_16:0_14:0)	782.7191 [M + NH_4_]^+^	2.71 (2.28–3.29)	3.49 (1.27–5.13)	0.6182
TG (45:1) TG (15:0_16:1_14:0) *	780.7089 [M + NH_4_]^+^	17.40 (9.96–20.78)	17.28 (7.56–35.83)	0.7243
TG (45:2) TG (15:0_16:1_14:1) *	778.6920 [M + NH_4_]^+^	15.10 (8.58–17.45)	11.07 (5.15–18.75)	0.6706
TG (46:1) TG (12:0_16:0_18:1)	794.7244 [M + NH_4_]^+^	84.73 (46.54–112.9)	35.46 (16.16–80.47)	0.1220
TG (46:2) TG (12:0_16:1_18:1) *	792.7077 [M + NH_4_]^+^	107.0 (57.19–131.1)	55.54 (36.47–129.3)	0.4285
TG (46:3) TG (12:0_16:1_18:2) *	790.6927 [M + NH_4_]^+^	63.47 (36.38–77.43)	24.74 (12.94–44.32)	0.0522
TG (46:4) TG (12:0_16:1_18:3) *	788.6762 [M + NH_4_]^+^	51.18 (25.02–53.76)	17.87 (8.62–38.09)	0.0522
TG (48:0) TG (14:0_16:0_18:0)	824.7640 [M + NH_4_]^+^	4.18 (2.77–6.00)	3.17 (1.81–5.23)	0.4285
TG (48:1) TG (14:0_16:0_18:1) *	822.7535 [M + NH_4_]^+^	25.14 (15.97–38.51)	15.91 (10.74–29.56)	0.1639
TG (48:2) TG (14:0_16:1_18:1) *	820.7704 [M + NH_4_]^+^	56.29 (32.47–73.22)	31.56 (20.61–62.97)	0.2761
TG (48:3) TG (14:0_16:1_18:2)	818.7230 [M + NH_4_]^+^	24.20 (15.44–32.25)	12.82 (6.57–21.61)	0.0627
TG (48:4) TG (14:1_16:1_18:2)	816.7074 [M + NH_4_]^+^	26.93 (14.65–29.98)	7.93 (6.63–12.28)	0.0288
TG (48:5) TG (14:1_16:1_18:3)	814.6906 [M + NH_4_]^+^	29.13 (21.40–34.61)	23.58 (13.62–28.86)	0.2150
TG (49:0) TG (15:0_16:0_18:0)	838.7803 [M + NH_4_]^+^	1.14 (0.88–1.20)	1.59 (1.07–2.75)	0.0627
TG (49:1) TG (15:0_16:0_18:1) *	836.7711 [M + NH_4_]^+^	5.24 (4.56–5.84)	8.21 (6.58–13.38)	0.0029
TG (49:2) TG (15:0_16:1_18:1) *	834.7572 [M + NH_4_]^+^	5.69 (4.20–6.36)	9.58 (7.32–14.53)	0.0089
TG (49:3) TG (15:0_16:1_18:2)	832.7411 [M + NH_4_]^+^	1.53 (1.25–1.69)	3.56 (1.79–4.47)	0.0147
TG (50:0) TG (16:0_16:0_18:0)*	852.7946 [M + NH_4_]^+^	2.73 (2.23–4.05)	3.01 (2.40–5.89)	0.5678
TG (50:1) TG (16:0_16:0_18:1) *	850.7845 [M + NH_4_]^+^	16.55 (12.76–26.79)	16.69 (10.79–37.13)	0.7243
TG (50:2) TG (16:0_16:1_18:1) *	848.7713 [M + NH_4_]^+^	49.71 (31.73–62.43)	49.90 (32.06–83.68)	0.4727
TG (50:3) TG(18:1_16:1_16:1) *	846.7565 [M + NH_4_]^+^	26.49 (15.37–30.01)	36.82 (15.44–46.66)	0.1883
TG (50:4) TG(18:1_16:1_16:1)	844.7411 [M + NH_4_]^+^	14.18 (7.82–16.43)	12.29 (3.22–16.46)	0.9502
TG (51:0) TG(18:0_15:0_18:0)	866.8108 [M + NH_4_]^+^	0.66 (0.47–0.79)	1.78 (1.35–3.01)	<0.0001
TG (51:1) TG (18:1_15:0_18:0) *	864.8004 [M + NH_4_]^+^	3.63 (2.40–4.76)	9.92 (6.16–16.59)	0.0005
TG (51:2) TG (18:1_15:0_18:1) *	862.7869 [M + NH_4_]^+^	7.62 (5.49–10.86)	22.02 (10.42–33.17)	0.0039
TG (51:3) TG (18:2_15:0_18:1) *	860.7725 [M + NH_4_]^+^	3.74 (2.37–5.12)	12.12 (4.02–15.19)	0.0115
TG (51:4) TG (18:2_15:0_18:2)	858.7570 [M + NH_4_]^+^	0.71 (0.37–0.95)	2.73 (0.52–3.33)	0.0288
TG(52:0) TG (16:0_18:0_18:0)	880.8267 [M + NH_4_]^+^	2.21 (1.13–3.21)	3.74 (2.92–6.74)	0.0232
TG(52:1) TG (16:0_18:0_18:1) *	878.8203 [M + NH_4_]^+^	11.37 (4.98–16.74)	20.06 (12.26–35.94)	0.0431
TG (52:2) TG (16:0_18:1_18:1) *	876.8029 [M + NH_4_]^+^	66.36 (44.57–104.40)	90.88 (53.70–158.70)	0.2150
TG (52:3) TG (16:1_18:1_18:1) *	874.7866 [M + NH_4_]^+^	50.73 (31.83–70.93)	70.95 (33.80–108.2)	0.2150
TG (52:4) TG(16:1_18:1_18:2) *	872. 7717 [M + NH_4_]^+^	23.66 (13.66–32.86)	37.68 (11.42–47.29)	0.1883
TG (52:5) TG(16:1_18:2_18:2)	870. 7572 [M + NH_4_]^+^	8.53 (4.55–11.06)	11.29 (1.74–15.65)	0.2761
TG (52:6) TG(16:1_18:2_18:3)	868. 7391 [M + NH_4_]^+^	2.03 (0.96–2.60)	1.69 (0.36–2.50)	0.7796
TG (53:0) TG(18:0_15:0_20:0)	894. 8438 [M + NH_4_]^+^	0.27 (0.20–0.31)	0.74 (0.36–1.03)	0.0052
TG (53:1) TG(18:0_15:0_20:1)	892. 8306 [M + NH_4_]^+^	0.99 (0.62–1.38)	5.57 (2.06–7.62)	0.0011
TG (53:2) TG (18:1_15:0_20:1) *	890.8179 [M + NH_4_]^+^	3.46 (2.44–5.32)	17.79 (5.74–23.76)	0.0011
TG (53:3) TG (18:2_15:0_20:1) *	888.8028 [M + NH_4_]^+^	3.40 (2.08–4.95)	15.12 (4.49–19.56)	0.0039
TG (54:2) TG (18:0_18:1_18:1) *	904.8342 [M + NH_4_]^+^	9.79 (6.34–16.05)	27.61 (10.16–45.41)	0.0147
TG (54:3) TG (18:1_18:1_18:1) *	902.8177 [M + NH_4_]^+^	19.24 (10.29–27.89)	33.89 (17.77–61.75)	0.0627
TG (54:4) TG (18:1_18:2_18:1)	900.8019 [M + NH_4_]^+^	17.58 (9.56–24.55)	26.85 (11.40–45.35)	0.1883
TG (54:5) TG (18:1_18:2_18:2)	898.7882 [M + NH_4_]^+^	14.80 (7.32–19.47)	23.47 (6.02–32.41)	0.1220
TG (56:0) TG (18:0_18:0_20:0)	936.8894 [M + NH_4_]^+^	0.20 (0.17–0.35)	0.31 (0.22–0.43)	0.1419
TG (56:1) TG (18:1_18:0_20:0)	934.8748 [M + NH_4_]^+^	0.49 (0.38–0.59)	0.94 (0.58–1.34)	0.0011
TG (56:2) TG (18:1_18:1_20:0)	932.8630 [M + NH_4_]^+^	0.96 (0.56–1.27)	2.20 (0.98–2.51)	0.0232
TG (56:3) TG (18:0_18:0_20:3)	930.8506 [M + NH_4_]^+^	1.18 (0.61–1.69)	2.24 (0.50–2.94)	0.1419
TG (56:4) TG (18:1_18:0_20:3)	928.8312 [M + NH_4_]^+^	1.43 (0.85–2.07)	1.40 (0.54–2.15)	0.9999
TG (56:5) TG (18:1_18:1_20:3)	926.8170 [M + NH_4_]^+^	3.49 (2.11–6.27)	3.88 (0.94–5.47)	0.7796
Diacylglyceride (DG)		35.91 (27.26–41.18) µM	29.31 (24.84–49.76) µM	0.7796
DG (24:0) DG (12:0_12:0) *	474.4160 [M + NH_4_]^+^	3.34 (2.77–5.00)	1.19 (0.75–2.32)	0.0015
DG (26:0) DG (12:0_14:0) *	502.4471 [M + NH_4_]^+^	3.96 (3.33–5.29)	1.90 (1.62–3.56)	0.0185
DG (28:0) DG (14:0_14:0) *	530.4785 [M + NH_4_]^+^	2.11 (1.10–3.17)	0.61 (0.35–0.84)	0.0011
DG (28:1) DG (14:0_14:1) *	511.4367 [M + NH_4_]^+^	1.66 (1.03–1.89)	1.16 (0.78–1.74)	0.3867
DG (28:2) DG (14:1_14:1) *	526.4471 [M + NH_4_]^+^	1.50 (0.99–2.37)	0.84 (0.68–1.82)	0.1419
DG (30:0) DG (14:0_16:0)	558.5097 [M + NH_4_]^+^	0.75 (0.56–1.54)	0.30 (0.17–0.50)	0.0011
DG (30:1) DG (14:0_16:1) *	556.4946 [M + NH_4_]^+^	1.76 (1.42–3.02)	1.17 (0.74–1.66)	0.0522
DG (30:2) DG (14:1_16:1)	554.4784 [M + NH_4_]^+^	0.60 (0.31–0.88)	3.51 (2.43–5.97)	<0.0001
DG (32:0) DG (16:0_16:0)	586.5417 [M + NH_4_]^+^	0.83 (0.48–1.46)	0.58 (0.27–0.91)	0.1419
DG (32:1) DG (16:0_16:1)	584.5247 [M + NH_4_]^+^	1.54 (1.07–1.92)	1.48 (1.07–2.28)	0.8928
DG (32:2) DG (16:1_16:1)	582.5090 [M + NH_4_]^+^	0.16 (0.11–0.30)	0.67 (0.26–1.24)	0.0015
DG (34:0) DG (16:0_18:0)	614.5728 [M + NH_4_]^+^	0.49 (0.27–0.63)	0.69 (0.32–1.03)	0.1883
DG (34:1) DG (16:0_18:1) *	612.5570 [M + NH_4_]^+^	2.18 (1.68–3.37)	5.34 (3.34–6.55)	0.0089
DG (34:2) DG (16:0_18:2)	610.5403 [M + NH_4_]^+^	0.52 (0.27–0.70)	2.13 (1.38–3.95)	0.0001
DG (34:3) DG (16:1_18:2)	608.5253 [M + NH_4_]^+^	0.56 (0.32–0.96)	1.06 (0.61–1.73)	0.0288
DG (36:0) DG (18:0_18:0)	642.6010 [M + NH_4_]^+^	0.12 (0.07–0.19)	0.04 (0.04–0.06)	0.0005
DG (36:1) DG (18:0_18:1)	640.5874 [M + NH_4_]^+^	0.48 (0.29–0.62)	1.05 (0.63–1.19)	0.0029
DG (36:2) DG (18:1_18:1) *	638.5731 [M + NH_4_]^+^	0.60 (0.31–0.88)	3.51 (2.43–5.97)	<0.0001
DG (36:3) DG (18:1_18:2) *	636.5557 [M + NH_4_]^+^	0.44 (0.25–0.53)	1.56 (0.75–2.56)	0.0003
DG (36:4) DG (18:2_18:2)	634.5397 [M + NH_4_]^+^	1.01 (0.58–1.36)	1.46 (1.10–2.43)	0.0288
DG (36:5) DG (18:2_18:3)	632.5248 [M + NH_4_]^+^	0.22 (0.14–0.58)	0.54 (0.39–0.91)	0.0288
DG (38:0) DG (18:0_20:0)	670.6335 [M + NH_4_]^+^	0.16 (0.11–0.28)	0.03 (0.02–0.04)	<0.0001
DG (38:1) DG (18:1_20:0)	668.6169 [M + NH_4_]^+^	0.35 (0.21–0.50)	0.09 (0.07–0.13)	0.0003
DG (38:2) DG (18:1_20:1)	666.6041 [M + NH_4_]^+^	0.07 (0.06–0.11)	0.06 (0.05–0.09)	0.7799
DG (38:3) DG (18:0_20:3)	664.5797 [M + NH_4_]^+^	0.02 (0.01–0.03)	0.03 (0.02–0.05)	0.2150
DG (38:4) DG (18:1_20:3) *	645.5496 [M + NH_4_]^+^	3.37 (2.13–4.69)	1.07 (0.65–1.29)	0.0021
DG (38:5) DG (18:3_20:2)	660.5046 [M + NH_4_]^+^	0.55 (0.39–0.84)	0.94 (0.64–1.23)	0.0354
DG (38:6) DG (18:2_20:4)	658.5450 [M + NH_4_]^+^	1.32 (0.76–1.62)	0.57 (0.46–0.77)	0.0089
DG (40:0) DG (20:0_20:0)	698.6656 [M + NH_4_]^+^	0.26 (0.18–0.43)	0.07 (0.03–0.08)	0.0001
DG (40:1) DG (20:0_20:1)	696.6494 [M + NH_4_]^+^	0.41 (0.28–0.61)	0.07 (0.05–0.13)	0.0003
Phosphatidylcholine (PC)		566.6 (411.7–752.5) µM	528.1 (442.4–900.3) µM	0.9502
PC (28:0) PC (10:0_18:0)	678.5066 [M + H]^+^	7.21 (3.75–9.03)	0.63 (0.28–1.12)	<0.0001
PC (30:0) PC (14:0_16:0)	706.5389 [M + H]^+^	31.17 (19.97–41.88)	5.50 (2.60–8.08)	0.0001
PC (30:1) PC (14:0_16:1)	704.5225 [M + H]^+^	2.17 (1.36–2.99)	0.39 (0.22–0.72)	0.0001
PC (32:0) PC (16:0_16:0) *	734.5692 [M + H]^+^	46.18 (34.93–54.82)	30.19 (21.18–42.55)	0.0887
PC (32:1) PC (16:0_16:1)	732.5541 [M + H]^+^	21.33 (16.75–27.15)	14.54 (8.73–21.53)	0.0354
PC (32:2) PC (16:1_16:1)	730.6375 [M + H]^+^	3.83 (1.95–4.93)	0.98 (0.37–1.41)	0.0003
PC (34:0) PC (16:0_18:0)	762.6004 [M + H]^+^	3.83 (1.95–4.93)	0.98 (0.37–1.41)	0.8357
PC (34:1) PC (16:0_18:1) *	760.5851 [M + H]^+^	73.30 (57.07–101.9)	126.4 (75.15–177.4)	0.0522
PC (34:2) PC (16:0_18:2)	758.5682 [M + H]^+^	95.03 (86.41–110.2)	93.10 (60.65–139.5)	0.8357
PC (34:3) PC (16:1_18:2) and PC (16:0_18:3)	756.5528 [M + H]^+^	4.42 (3.94–5.41)	4.41 (2.49–5.96)	0.7796
PC (36:0) PC (18:0_18:0) and PC (16:0_20:0)	790.6289 [M + H]^+^	1.22 (0.19–2.19)	1.17 (0.47–3.39)	0.7243
PC (36:1) PC (18:0_18:1) *	788.6159 [M + H]^+^	32.11 (16.49–43.44)	35.87 (26.60–75.70)	0.2443
PC (36:2) PC (18:0_18:2) and PC (18:1_18:1) *	786.6017 [M + H]^+^	99.91 (67.75–129.0)	116.1 (79.61–168.3)	0.1883
PC (36:3) PC (18:1_18:2)	784.5837 [M + H]^+^	50.31 (46.37–69.53)	75.70 (49.40–124.0)	0.1419
PC (36:4) PC (16:0_20:4) and PC (16:1_20:3)	782.5694 [M + H]^+^	52.57 (41.65–63.18)	45.34 (36.53–85.27)	0.5191
PC (36:5) PC (16:1_20:4) and PC (18:2_18:3) *	780.5540 [M + H]^+^	2.74 (2.56–3.29)	2.47 (1.54–4.46)	0.6706
Phosphatidylethanolamine (PE)		1674 (932.8–2653) µM	1325 (499.3–2298) µM	0.6490
PE (32:1) PE (16:0_16:1)	690.5049 [M + H]^+^	26.72 (13.64–35.02)	6.60 (3.40–13.52)	0.0057
PE (34:1) PE (16:0_18:1)	718.5385 [M + H]^+^	76.78 (31.67–96.56)	35.92 (16.94–47.93)	0.2092
PE (34:2) PE (16:0_18:2)	716.5209 [M + H]^+^	116.7 (72.99–187.3)	51.44 (20.70–153.2)	0.0943
PE (34:3) PE (16:1_18:2)	714.5044 [M + H]^+^	12.23 (10.60–28.92)	9.25 (2.37–22.0)	0.2404
PE (36:0) PE (18:0_18:0)	748.5861 [M + H]^+^	46.42 (27.28–56.99)	58.47 (26.58–77.00)	0.4374
PE (36:1) PE (16:0_20:1) *	746.5699 [M + H]^+^	209.5 (123.0–367.9)	236.1 (110.7–358.8)	0.8872
PE (36:2) PE (16:0_20:2) *	744.5538 [M + H]^+^	718.5 (317.5–1025)	588.2 (200–835.9)	0.4890
PE (36:3) PE (18:1_18:2)	742.5379 [M + H]^+^	227.2 (156.3–375.2)	260.4 (85.39–677.6)	0.6447
PE (36:4) PE (18:2_18:2) *	740.5211 [M + H]^+^	167.2 (119.9–306.7)	63.09 (19.33–157.1)	0.0534
PE (36:5) PE (18:2_18:3) and PE (16:1_20:4)	738.5063 [M + H]^+^	21.69 (14.80–40.73)	6.56 (3.07–21.5)	0.0434
PE (38:0) PE (18:0_20:0)	776.6201 [M + H]^+^	8.22 (2.64–13.90)	22.22 (9.07–31.58)	0.0433
PE (36:4p) PE (18:3_P-18:1) *	722.5137 [M + H]^+^	3.19 (2.58–3.81)	1.13 (0.95–1.31)	<0.0001
PE (40:4) (16:0_16:0) *	734.5678 [M + FA-H]^-^	3.40 (2.91–5.07)	1.21 (0.76–1.98)	<0.0001
Ceramides (Cer)		67.46 (48.24–85.66) µM	60.14 (36.99–93.63) µM	0.7243
Cer 14:0	492.4778 [M + H]^+^	1.34 (0.61–2.03)	0.31 (0.17–0.78)	0.0015
Cer 16:0	520.5098 [M + H]^+^	11.23 (6.29–16.64)	9.50 (4.13–17.65)	0.6706
Cer 16:1	518.4925 [M + H]^+^	2.85 (1.36–4.10)	4.42 (1.62–5.06)	0.3473
Cer 18:0	548.5404 [M + H]^+^	2.09 (1.19–2.62)	3.36 (1.91–4.47)	0.0627
Cer 18:1	546.5239 [M + H]^+^	0.25 (0.15–0.48)	1.57 (0.62–2.36)	0.0015
Cer 20:0	576.5718 [M + H]^+^	0.31 (0.21–0.39)	0.66 (0.40–0.92)	0.0039
Cer 20:1	574.5548 [M + H]^+^	0.23 (0.15–0.26)	0.42 (0.23–0.76)	0.0147
Cer 22:0	604.5019 [M + H]^+^	0.32 (0.24–0.62)	0.41 (0.17–0.77)	0.9502
Cer 22:1	602.5855 [M + H]^+^	4.80 (2.81–6.31)	5.68 (3.36–11.36)	0.3473
Cer 24:0	632.6333 [M + H]^+^	5.84 (4.85–7.45)	6.10 (3.92–7.79)	0.9999
Cer 24:1	630.6180 [M + H]^+^	37.69 (26.92–48.59)	23.43 (14.44–46.03)	0.1639
Sphingomyelines (SM)		146.8 (128.3–193.7) µM	136.4 (108.3–160.8) µM	0.4285
SM 12:0	647.5114 [M + H]^+^	1.49 (0.99–2.11)	0.40 (0.19–0.66)	0.0011
SM 14:0	675.5430 [M + H]^+^	4.70 (3.81–5.94)	1.49 (0.77–2.38)	0.0003
SM 16:0	703.5744 [M + H]^+^	68.03 (59.02–84.67)	55.94 (39.94–71.18)	0.1883
SM 16:1	701.5587 [M + H]^+^	7.27 (6.16–8.54)	5.62 (3.28–8.51)	0.3867
SM 18:0	731.6054 [M + H]^+^	2.09 (1.20–3.07)	11.42 (8.40–13.32)	<0.0001
SM 18:1	729.5896 [M + H]^+^	1.91 (1.67–2.22)	7.61 (4.83–8.55)	0.0005
SM 20:1	757.6215 [M + H]^+^	0.38 (0.10–0.69)	0.63 (0.44–1.12)	0.1220
SM 22:0	787.6611 [M + H]^+^	6.21 (3.27–11.11)	5.28 (1.01–8.82)	0.5678
SM 22:1	785.6531 [M + H]^+^	4.69 (3.38–6.08)	6.54 (5.23–7.92)	0.1044
SM 24:0	815.6954 [M + H]^+^	11.82 (8.55–20.29)	7.42 (6.68–12.29)	0.0749
SM 24:1	813.6865 [M + H]^+^	37.35 (21.37–46.82)	25.44 (21.22–38.16)	0.5191
SM 26:0	843.7257 [M + H]^+^	5.93 (2.90–7.89)	2.79 (0.46–3.55)	0.0288
SM 26:1	841.7096 [M + H]^+^	1.97 (1.01–3.68)	1.05 (0.38–1.75)	0.1883

Based on LipidMAPS nomenclature, we used “_” in case of a putative acyl chain position on the glycerol.

**Table 3:** Concentrations (µM, except for SM 20:1 expressed in nM) of sphingolipids (SPLs) in blood of male and female offspring at day 25 of lactation period and suckled by dams fed control or western diet.

Lipid Species (µM)				Groups			2-Way ANOVA		
	WD	C	WD-F	WD-M	C-F	C-M	Global Effects		
n	11	11	6	5	5	6	Inter	Diet	Sex
Cer 12:0	0.157 ± 0.007	0.143 ± 0.007	0.152 ±0.008^,^	0.164 ± 0.012	0.152 ± 0.009	0.135 ± 0.011	0.160	0.191	0.791
Cer 16:0	0.194 ± 0.018	0.214 ± 0.008	0.195 ±0.032	0.191 ± 0.015	0.227 ± 0.014	0.204 ± 0.007	0.658	0.295	0.518
Cer 18:0	0.627 ± 0.138	0.395 ± 0.031	0.742 ± 0.235	0.511 ± 0.123	0.409 ± 0.043	0.384 ± 0.048	0.525	0.145	0.425
Cer 18:1)	0.008 ± 0.0004	0.008 ± 0.0002	0.009 ± 0.0006	0.008 ± 0.0005	0.009 ± 0.0003	0.009 ± 0.0003	0.548	0.393	0.559
Cer 22:0	0.846 ± 0.116	0.720 ± 0.053	0.925 ± 0.156	0.751 ± 0.182	0.819 ± 0.088	0.637 ± 0.044	0.973	0.182	0.402
Cer 24:0	4.107 ± 0.335	3.280 ± 0.156 ^a^	4.639 ± 0.447 ^$$$^	3.468 ± 0.360	3.727 ± 0.189	2.908 ± 0.083 **	<0.001	<0.001	0.0006
Cer 24:1)	1.290 ± 0.169	0.996 ± 0.047	1.392 ± 0.230	1.169 ± 0.267	1.091 ± 0.076	0.918 ± 0.041	0.891	0.142	0.286
C18:S1P)	3.329 ± 0.191	3.202 ± 0.154	3.524 ± 0.249	3.096 ± 0.289	3.256 ± 0.277	3.157 ± 0.186	0.517	0.683	0.305
Hex-Cer 16:0	0.585 ± 0.087	0.538 ± 0.029	0.631 ± 0.152	0.529 ± 0.077	0.555 ± 0.056	0.524 ± 0.032	0.714	0.678	0.499
Hex-Cer 24:1	0.841 ± 0.088	0.780 ± 0.054	0.923 ± 0.131	0.743 ± 0.110	0.867 ± 0.093	0.707 ± 0.050	0.925	0.658	0.111
Hex-Cer 24:0)	0.225 ± 0.023	0.256 ± 0.015	0.266 ± 0.032	0.176 ± 0.016 * ^$^	0.281 ± 0.026	0.234 ± 0.013	0.378	0.141	0.009
Lacto-Cer 16:0	0.146 ± 0.031	0.109 ± 0.017	0.121 ± 0.033	0.176 ± 0.057	0.135 ± 0.032	0.087 ± 0.014	0.169	0.309	0.931
Lacto-Cer 24:1	0.049 ± 0.008	0.028 ± 0.006	0.057 ± 0.012 ^$^	0.040 ± 0.010	0.019 ± 0.008	0.035 ± 0.009	0.129	0.050	0.976
SM 12:0	0.001 ± 0.000	0.005 ± 0.000 ^c^	0.001 ± 0.000 ^$$$^	0.001 ± 0.000 ^$$$^	0.004 ± 0.007	0.005 ± 0.000	0.981	<0.001	0.751
SM 14:0	0.014 ± 0.000	0.038 ± 0.002 ^c^	0.015 ± 0.000 ^$$$^	0.013 ± 0.000 * ^$$$^	0.037 ± 0.003	0.038 ± 0.002	0.940	<0.001	0.405
SM 16:0	0.109 ± 0.006	0.270 ± 0.004 ^c^	0.101 ± 0.009 ^$$$^	0.117 ± 0.005 ^$$$^	0.267 ± 0.003	0.271 ± 0.008	0.424	<0.001	0.191
SM 18:0	0.109 ± 0.006	0.270 ± 0.004 ^c^	0.049 ± 0.007	0.041 ± 0.008 ^$$$^	0.072 ± 0.005	0.097 ± 0.008	0.038	<0.001	0.252
SM 20:0	0.019 ± 0.002	0.031 ± 0.001 ^c^	0.019 ± 0.003 ^$$^	0.017 ± 0.002 ^$$$^	0.032 ± 0.001	0.030 ± 0.002	0.288	<0.001	0.875
SM 22:0	0.064 ± 0.009	0.110 ± 0.005 ^b^	0.071 ± 0.015 ^$^	0.055 ± 0.011 ^$$^	0.110 ± 0.010	0.111 ± 0.004	0.465	0.004	0.504
SM 24:0)	0.081 ± 0.014	0.140 ± 0.008 ^b^	0.095 ± 0.021	0.065 ± 0.016 ^$^	0.143 ± 0.018	0.138 ± 0.008	0.456	0.002	0.305
SM 18:1	0.046 ± 0.005	0.086 ± 0.006 ^c^	0.011 ± 0.008 ^$$^	0.010 ± 0.000 ^$$$^	0.015 ± 0.000	0.017 ± 0.001	0.127	<0.001	0.631
SM 20:1 (nM)	0.477 ± 0.045	1.094 ± 0.065 ^c^	0.450 ± 0.058 ^$$$^	0.509 ± 0.075 ^$$$^	0.983 ± 0.072	1.186 ± 0.091	0.357	<0.001	0.104
SM 24:1	0.074 ± 0.008	0.133 ± 0.007 ^c^	0.079 ± 0.014 ^$^	0.067 ± 0.009 ^$$$^	0.129 ± 0.014	0.136 ± 0.006	0.408	<0.001	0.839
Cer total	7.230 ± 0.722	5.758 ± 0.261	8.036 ± 1.027	6.263 ± 0.924	6.434 ± 0.352	5.195 ± 0.168 **	0.719	0.083	*0.0533*
LC-Cer total	0.821 ± 0.151	0.609 ± 0.038	0.920 ± 0.259	0.702 ± 0.137	0.635 ± 0.056	0.588 ± 0.0540	0.663	0.302	0.516
Very LC-Cer total	0.821 ± 0.151	0.609 ± 0.038	0.60 ± 0.09	0.60 ± 0.09	1.65 ± 0.25	0.95 ± 0.26 **	0.671	0.0434	0.0197
Hexo/Lacto-Cer total	11.100 ± 1.080	9.768 ± 0.472	12.430 ± 1.573	9.510 ± 1.254	9.622 ± 1.153	8.889 ± 0.373	0.661	0.327	0.041
SM total	0.419 ± 0.043	0.830 ± 0.026 ^c^	0.442 ± 0.070 ^$$$^	0.391 ± 0.052 ^$$$^	0.809 ± 0.047	0.847 ± 0.030	0.406	< 0.001	0.860

Values were mean ± SEM and were analyzed with two-way ANOVA with dam’s diet and offspring sex factors. ANOVA was followed by Sidak’s multiple comparisons post-hoc test for comparisons between both diet groups for each sex with $, *p* <0.05, $$, *p* <0.01 and $$$, *p* <0.001 significantly different. For each biomarker, values of p-values (assessed by Mann–Whitney U test) between “western diet” and “control diet” groups, regardless of sex group, were reported with a, b, c significantly different; *p* < 0.05, *p* < 0.01 or *p* < 0.001, respectively. Values of *p*-values (assessed by Mann–Whitney U test) between “female” and “male” groups considering each diet group, were reported with *, *p*< 0.05, **, *p*< 0.01 significantly different. LC-Cer: Long chain ceramide (from 16 to 20 carbons) and Very LC-Cer that contains at least 22 carbons.

**Table 4:** General diet in compositions (expressed in % of kcal).

Nutriments	Control Diet (CD)	Western Diet (WD)
Cellulose	4.22	5.00
Sucrose	0.00	29.45
Beef fat	4.22	20.00
C8-C12:0	0.01	0.06
C14:0	0.12	0.67
C16:0	1.02	5.20
C16:1	0.12	0.63
C18:0	0.79	4.22
C18:1	1.53	7.59
C18:2	0.55	1.23
C18:3	0.04	0.17
C20-C22	0.04	0.22
Casein	18.55	22.00
Dextrose	8.43	10.00
Starch	57.50	5.00
Total Energy (kcal/100 g)	377.1	466.3

## Supplementary Materials

The following are available online at https://www.mdpi.com/1422-0067/21/15/5428/s1, Table S1: Profiles of triglycerides (TGs) (expressed in % of total TG) in blood of male and female offspring at day 25 of life and suckled by dams fed Control or Western diet. Figure S1: PLS-DA score plot for 25 data points (SLs), reveals natural clusters for all the male (MC, MWD) and female (FC, FWD) 25 days old-offspring.
