# Supplementary material for: Moderate High Caloric Maternal Diet Impacts Dam Breast Milk Metabotype and Offspring Lipidome in a Sex-Specific Manner
Source: Int J Mol Sci. 2020 Jul 30;21(15):5428. doi: 10.3390/ijms21155428 (PMC7432416; doi:10.3390/ijms21155428)
Supplement: Supplementary file 1 [file ijms-21-05428-s001.pdf]

Supplementary Table

Table S1. Profiles of triglycerides (TGs) (expressed in % of total TG) in blood of male and female offspring at day 25 of life and suckled by dams fed control or western diet.

Lipid Species	WD	C	WD-F	WD-M	Groups C-F	C-M	2-way ANOVA Global Effects		
**n**	**11**	11	6	5	5	6	Inter	Diet	Sex
TG(34:0) (C10:0_C10:0_C14:0)	0.011 ± 0.002	0.013 ± 0.002	0.013 ± 0.003 ′	0.008 ± 0.001	0.011 ± 0.003	0.016 ± 0.003	0.071	0.287	0.996
TG(36:0) (C10:0_C12:0_C14:0)	0.030 ± 0.005	0.036 ± 0.003	0.037 ± 0.009	0.022 ± 0.004	0.033 ± 0.002	0.039 ± 0.006	0118	0.336	0.508
TG(38:0) (C10:0_C12:0_C16:0)	0.027 ± 0.005	0.027 ± 0.003	0.035 ± 0.007	0.018 ± 0.005	0.027 ± 0.004	0.027 ± 0.005	0.525	0.145	0.425
TG(40:0) (C10:0_C14:0_C16:0)	0.018 ± 0.005	0.021 ± 0.003	0.026 ± 0.007	0.009 ± 0.003 [*]	0.017 ± 0.003	0.024 ± 0.006	0.037	0.587	0.359
TG(42:0) (C10:0_C14:0_C18:0)	0.066 ± 0.013	0.074 ± 0.013	0.089 ± 0.017	0.038 ± 0.009 *	0.091 ± 0.025	0.060 ± 0.011	0.547	0.508	0.025
TG(42:1) (C10:0_C14:1_C18:0)	0.041 ± 0.006	0.056 ± 0.006	0.048 ± 0.009	0.031 ± 0.006	0.056 ± 0.006	0.056 ± 0.010	0.393	0.078	0.331
TG(42:2) (C10:0_C14:1_C18:1)	0.014 ± 0.005 [b]	0.031 ± 0.004	0.020 ± 0.008	0.006 ± 0.002 [$]	0.030 ± 0.009	0.032 ± 0.005	0.252	0.011	0.354
TG(43:0) (C14:0_C15:0_C14:0)	0.049 ± 0.011	0.063 ± 0.008	0.064 ± 0.017	0.030 ± 0.007	0.059 ± 0.009	0.066 ± 0.012	0.130	0.246	0.286
TG(43:1) (C14:0_C15:0_C14:1)	0.021 ± 0.005	0.028 ± 0.004	0.028 ± 0.008	0.014 ± 0.003	0.025 ± 0.004	0.030 ± 0.007	0.149	0.300	0.471
TG(43:2) (C14:1_C15:0_C14:1)	0.021 ± 0.006 [b]	0.045 ± 0.006	0.029 ± 0.011	0.009 ± 0.002 [$]	0.043 ± 0.012	0.047 ± 0.008	0.234	0.014	0.423
TG(44:0) (C12:0_C14:0_C18:0)	0.225 ± 0.023	0.256 ± 0.015	0.266 ± 0.032	0.176 ± 0.016	0.281 ± 0.026	0.234 ± 0.013	0.276	0.779	0.060
TG(44:1) (C12:0_C14:1_C18:0)	0.195 ± 0.032	0.238 ± 0.024	0.240 ± 0.051	0.141 ± 0.021	0.238 ± 0.041	0.238 ± 0.032	0.221	0.246	0.228
TG(44:2) (C12:0_C14:1_C18:1)	0.038 ± 0.003 [a]	0.051 ± 0.005	0.040 ± 0.006 [$]	0.036 ± 0.003	0.059 ± 0.009	0.044 ± 0.006	0.377	0.047	0.140

TG(45:0) (C16:0_C15:0_C14:0)	0.133 ± 0.038	0.207 ± 0.032 [c]	0.203 ± 0.052	0.050 ± 0.028 *	0.228 ± 0.063	0.189 ± 0.034	0.236	0.092	0.054
TG(45:1) (C16:0_C15:0_C14:1)	0.195 ± 0.035	0.276 ± 0.031[c]	0.230 ± 0.059	0.154 ± 0.027	0.254 ± 0.048	0.294 ± 0.043	0.237	0.104	0.705
TG(45:2) (C16:1_C15:0_C14:1)	0.068 ± 0.011	0.080 ± 0.007	0.083 ± 0.017	0.049 ± 0.007	0.075 ± 0.008	0.084 ± 0.011	0.054	0.178	0.514
TG(46:1) (C16:0_C16:0_C14:1)	0.578 ± 0.118	0.704 ± 0.094	0746 ± 0.189	0.376 ± 0.066	0.698 ± 0.162	0709 ± 0.122	0.213	0.347	0.241
TG(46:2) (C16:1_C16:0_C14:1)	0.326 ± 0.043	0.376 ± 0.035	0.385 ± 0.070 $$	0.256 ± 0.021	0.366 ± 0.055	0.384 ± 0.051	0.195	0.332	0.319
TG(46:3) (C16:1_C16:1_C14:1)	0.024 ± 0.002	0.025 ± 0.003 [b]	0.024 ± 0.003	0.025 ± 0.003	0.020 ± 0.002	0.029 ± 0.005	0.311	0.925	0.181
TG(46:4) (C14:0_C18:3_C14:1)	0.311 ± 0.096	0.300 ± 0.049	0.438 ± 0.154	0.157 ± 0.065	0.307 ± 0.107	0.295 ± 0.037	0.214	0.976	0.177
TG(48:0) (C14:0_C18:0_C16:0)	0.059 ± 0.017	0.070 ± 0.016[c]	0.083 ± 0.027	0.030 ± 0.008	0.076 ± 0.035	0.065 ± 0.009	0.376	0.557	0.179
TG(48:1) (C14:1_C18:0_C16:0)	0.553 ± 0.158	0.689 ± 0.107	0.802 ± 0.246	0.253 ± 0.077$	0.635 ± 0.195	0.735 ± 0.125	0.088	0.392	0.226
TG(48:2) (C14:1_C18:0_C16:1)	0.528 ± 0.042	0.653 ± 0.053	0.507 ± 0.056	0.553 ± 0.069	0.685 ± 0.087	0.626 ± 0.071	0.469	0.093	0.922
TG(48:3) (C14:1_C18:1_C16:1)	0.171 ± 0.027	0.201 ± 0.023	0.216 ± 0.041	0.117 ± 0.014	0.186 ± 0.039	0.214 ± 0.029	0.076	0.328	0.302
TG(48:5) (C14:1_C18:3_C16:1)	3.999 ± 0.492	3.652 ± 0.169	4.902 ± 1.109	2.915 ± 0.639	3.794 ± 0.299	3.533 ± 0.392	0.249	0.739	0.138
TG(49:0) (C15:0_C18:0_C16:0)	0062 ± 0.008	0.083 ± 0.009	0.072 ± 0.014	0.050 ± 0.005	0.073 ± 0.017	0.091 ± 0.012	0.149	0.125	0.866
TG(49:1) (C15:0_C18:1_C16:0)	0.716 ± 0.108	0.856 ± 0.084	0.879 ± 0.170	0.520 ± 0.062	0.822 ± 0.152	0.885 ± 0.100	0.128	0.259	0.278
TG(49:2) (C15:0_C18:1_C16:1)	0.610 ± 0.076	0.783 ± 0.086	0.676 ± 0.136	0.531 ± 0.047	0.719 ± 0.168	0.836 ± 0.085	0.281	0.158	0.907
TG(49:3) (C15:0_C18:2_C16:1)	0.150 ± 0.015	0.179 ± 0.013	0.160 ± 0.027	0.139 ± 0.003	0.162 ± 0.019	0.193 ± 0.017	0.195	0.176	0.812
TG(50:0) (C16:0_C18:0_C16:0)	0.041 ± 0.011	0.043 ± 0.013	0.057 ± 0.016*	0.022 ± 0.008	0.046 ± 0.024	0.040 ± 0.017	0.410	0.827	0.238
TG(50:1) (C16:1_C18:0_C16:0)	2.098 ± 0.245 [a]	2.849 ± 0.110	2.656 ± 0.217**	1.427 ± 0.230 $$$	2.976 ± 0.189	2.743 ± 0.125	0.019	0.000	0.001

TG(50:2) (C16:1_C18:0_C16:1)	4.265 ± 0.161 [c]	5.496 ± 0.195	4.575 ± 0.180 [*$$]	3.893 ± 0.172 [$$$]	5.672 ± 0.359	5.349 ± 0.209	0.456	<0.001	0.047
TG(50:3) (C16:1_C18:1_C16:1)	1.229 ± 0.098	1.310 ± 0.061	1.053 ± 0.144 [*$$]	1.440 ± 0.034 [$$$]	1.349 ± 0.117	1.277 ± 0.063	0.0389	0.529	0.144
TG(50:4) (C16:1_C18:2_C16:1)	0.377 ± 0.066	0.431 ± 0.061	0.263 ± 0.078*	0.514 ± 0.079	0.472 ± 0.117	0.396 ± 0.063	0.070	0.596	0.319
TG(51:0) (C18:0_C15:0_C18:0)	0.047 ± 0.004	0.0470 ± 0.003	0.051 ± 0.006	0.042 ± 0.004	0.041 ± 0.005	0.041 ± 0.005	0.0764	0.905	0.917
TG(51:1) (C18:1_C15:0_C18:0)	0.497 ± 0.040	0.437 ± 0.024	0.585 ± 0.046 [*$]	0.390 ± 0.024	0.436 ± 0.041	0.438 ± 0.031	0.017	0.193	0.018
TG(51:2) (C18:1_C15:0_C18:1)	1.101 ± 0.056 [b]	0.824 ± 0.041	1.084 ± 0.072 [*$]	1.120 ± 0.097 [$]	0.804 ± 0.069	0.842 ± 0.054	0.988	0.001	0.619
TG(51:3) (C18:2_C15:0_C18:1)	0.606 ± 0.062	0.496 ± 0.039	0.497 ± 0.069 *	0.736 ± 0.078	0.465 ± 0.058	0.521 ± 0.054	0.181	0.075	0.037
TG(51:4) (C18:2_C15:0_C18:2)	0.162 ± 0.033	0.149 ± 0.023	0.107 ± 0.032 *	0.228 ± 0.050	0.143 ± 0.033	0.154 ± 0.036	0.165	0.618	0.096
TG(52:0) (C14:0_C18:0_C20:0)	0.257 ± 0.021	0.219 ± 0.026	0.309 ± 0.017 **	0.195 ± 0.013	0.213 ± 0.059	0.223 ± 0.014	0.053	0.100	0.275
TG(52:1) (C14:1_C18:0_C20:0)	2.208 ± 0.159	1.935 ± 0.251	2.610 ± 0.139 **	1.725 ± 0.062	1.710 ± 0.560	2.121 ± 0.074	0.027	0.362	0.390
TG(52:2) (C14:1_C18:1_C20:0)	16.15 ± 1.36	15.16 ± 0.66	15.94 ± 2.45	16.39 ± 1.08	15.23 ± 1.25	15.10 ± 0.75	0.856	0.539	0.922
TG(52:3) (C14:1_C18:2_C20:0)	16.71 ± 0.74	17.36 ± 0.22	16.05 ± 1.32	17.51 ± 0.35	17.22 ± 0.43	17.47 ± 0.23	0.447	0.476	0.287
TG(52:4) (C14:1_C18:2_C20:1)	10.28 ± 1.01 [a]	12.67 ± 0.63	8.87 ± 1.55	11.97 ± 0.85	12.67 ± 1.11	12.68 ± 0.82	0.199	0.067	0.195
TG(52:5) (C14:1_C18:3_C20:1)	2.101 ± 0.423	2.533 ± 0.377	1.318 ± 0.515	3.040 ± 0.427	2.636 ± 0.687	2.447 ± 0.452	0.086	0.499	0.162
TG(52:6) (C14:1_C18:3_C20:2)	0.067 ± 0.015	0.072 ± 0.018	0.049 ± 0.020	0.087 ± 0.022	0.078 ± 0.029	0.067 ± 0.024	0.318	0.876	0.581
TG(53:0) (C15:0_C18:0_C20:0)	0.021 ± 0.004	0.022 ± 0.004	0.024 ± 0.006	0.016 ± 0.006	0.018 ± 0.003	0.025 ± 0.006	0.210	0.846	0.925
TG(53:1) (C15:0_C18:1_C20:0)	0.067 ± 0.015 [a]	0.0718 ± 0.018	0.049 ± 0.020* [$$]	0.087 ± 0.022	0.078 ± 0.029	0.067 ± 0.024	0.023	0.016	0.064
TG(53:2) (C15:0_C18:1_C20:1)	1.459 ± 0.165 [c]	0.581 ± 0.034	1.407 ± 0.204 [$$]	1.522 ± 0.293 [$$]	0.578 ± 0.033	0.583 ± 0.060	0.758	<0.001	0.740

TG(53:3) (C15:0_C18:2_C20:1)	0.872 ± 0.117 ^{b}	0.452 ± 0.037	0.712 ± 0.131	1.065 ± 0.182 $^{\$\$}$	0.439 ± 0.034	0.463 ± 0.064	0.174	0.001	0.123
TG(53:4) (C15:0_C18:3_C20:1)	0.325 ± 0.066	0.242 ± 0.038	0.214 ± 0.073	0.459 ± 0.090 $^{\$\$}$	0.237 ± 0.054	0.246 ± 0.058	0.110	0.194	*0.086*
TG(54:0) (C16:0_C18:0_C20:0)	0.088 ± 0.009	0.097 ± 0.005	0.111 ± 0.007**	0.059 ± 0.005 $^{\$\$}$	0.099 ± 0.009	0.094 ± 0.004	0.004	0.116	0.0007
TG(54:1) (C16:1_C18:0_C20:0)	0.707 ± 0.065	0.606 ± 0.093	0.866 ± 0.047 $^{**\$}$	0.515 ± 0.056	0.464 ± 0.174	0.724 ± 0.077	0.005	0.330	0.645
TG(54:2) (C16:1_C18:0_C20:1)	5.243 ± 0.355 ^{b}	3.806 ± 0.181	5.349 ± 0.475	5.116 ± 0.589	3.828 ± 0.338	3.787 ± 0.210	0.821	0.003	0.748
TG(54:3) (C18:1_C18:1_C18:1)	9.782 ± 0.440 ^{a}	8.570 ± 0.199	9.912 ± 0.573	9.625 ± 0.750	8.514 ± 0.378	8.616 ± 0.216	0.706	0.029	0.858
TG(54:4) (C18:1_C18:2_C18:1)	7.927 ± 0.856	9.131 ± 0.512	8.059 ± 0.832	7.768 ± 1.727	9.191 ± 0.929	9.080 ± 0.620	0.933	0.262	0.851
TG(54:5) (C18:1_C18:3_C18:1)	2.219 ± 0.244 ^{a}	1.440 ± 0.199	2.052 ± 0.371	2.420 ± 0.320	1.456 ± 0.346	1.425 ± 0.255	0.547	0.026	0.616
TG(56:0) (C18:0_C18:0_C20:0)	0.017 ± 0.002	0.022 ± 0.003	0.023 ± 0.002	0.011 ± 0.002	0.019 ± 0.006	0.022 ± 0.002	0.054	0.259	0.204
TG(56:1) (C18:1_C18:0_C20:0)	0.068 ± 0.010 ^{b}	0.069 ± 0.009	0.066 ± 0.016	0.070 ± 0.013	0.069 ± 0.015	0.068 ± 0.012	0.906	0.959	0.917
TG(56:2) (C18:1_C18:1_C20:0)	0.677 ± 0.053	0.659 ± 0.050	0.649 ± 0.076	0.710 ± 0.076	0.613 ± 0.080	0.697 ± 0.065	0.878	0.753	0.351
TG(56:3) (C18:1_C18:2_C20:0)	1.281 ± 0.137	0.942 ± 0.116	1.121 ± 0.164	1.474 ± 0.213	0.851 ± 0.233	1.017 ± 0.104	0.606	0.057	0.164
TG(56:4) (C18:1_C18:2_C20:1)	0.381 ± 0.041	0.465 ± 0.053	0.411 ± 0.063	0.345 ± 0.052	0.484 ± 0.100	0.450 ± 0.059	0.823	0.219	0.486
TG(56:5) (C18:1_C18:3_C20:1)	1.454 ± 0.141 ^{b}	0.816 ± 0.130	1.507 ± 0.159	1.391 ± 0.264	0.928 ± 0.245	0.722 ± 0.136	0.823	0.006	0.432
SAT-TG	0.234 ± 0.008	0.233 ± 0.008	0.247 ± 0.009	0.218 ± 0.008	0.230 ± 0.016	0.235 ± 0.009	0.136	0.972	0.274
MC-SAT-TG	0.002 ± 0.000	0.002 ± 0.000	0.003 ± 0.000	0.001 ± 0.000	0.002 ± 0.000	0.002 ± 0.000	0.198	0.308	0.100
MUFA-TG	0.601 ± 0.003	0.595 ± 0.002	0.597 ± 0.005	0.607 ± 0.003	0.597 ± 0.005	0.594 ± 0.002	0.118	0.133	0.424
OA-TG	0.260 ± 0.003 ^{c}	0.240 ± 0.003	0.260 ± 0.005*$	0.260 ± 0.004** $	0.239 ± 0.006	0.241 ± 0.003	0.931	0.006	0.937
PUFA-n-6-TG	0.106 ± 0.005	0.111 ± 0.006	0.100 ± 0.006	0.113 ± 0.009	0.113 ± 0.012	0.110 ± 0.007	0.402	0.562	0.563
PUFA-n-3-TG	0.034 ± 0.002	0.029 ± 0.002	0.034 ± 0.003	0.033 ± 0.002	0.031 ± 0.004	0.028 ± 0.002	0.797	0.158	0.589
PUFA n-6/PUFA -n-3-TG	3.236 ± 0.237 ^{a}	3.853 ± 0.115	3.121 ± 0.404	3.374 ± 0.236	3.753 ± 0.149	3.935 ± 0.174	0.898	0.043	0.437

LC PUFA-TG	0.165 ± 0.007	0.172 ± 0.006	0.156 ± 0.009	0.175 ± 0.010	0.172 ± 0.011	0.171 ± 0.007	0.307	0.525	0.369
LA 18:2 n-6-TG	0.129 ± 0.008	0.141 ± 0.004	0.121 ± 0.012	0.140 ± 0.088	0.141 ± 0.007	0.142 ± 0.005	0.343	0.247	0.286
ALA 18:3 n-3_TG	0.035 ± 0.002	0.030 ± 0.002	0.035 ± 0.003	0.035 ± 0.002	0.031 ± 0.004	0.029 ± 0.002	0.703	0.138	0.695
EFA (LA+ALA) - TG	0.165 ± 0.007	0.172 ± 0.006	0.156 ± 0.009	0.175 ± 0.010	0.172 ± 0.011	0.171 ± 0.007	0.307	0.523	0.368
Unsaturated/saturated -TG	3.212 ± 0.126	3.211 ± 0.145	2.989 ± 0.138	3.481 ± 0.159	3.287 ± 0.270	3.147 ± 0.163	0.102	0.924	0.352
Total TGs (area 10^6)	3.908 ± 0.058	2.78 ± 0.026	3.25 ± 0.078	4.70 ± 0.080	2.91 ± 0.037	2.67 ± 0.039	0.193	0.074	0.350

Values were mean ± SEM and were analyzed with two-way ANOVA with dam's diet and offspring sex factors. ANOVA was followed by Sidak's multiple comparisons post-hoc test for comparisons between both diet groups for each sex with $, $p < 0.05$, $$, $p < 0.01$, and $$$, $p < 0.001$ significantly different. For each biomarker, values of p-values (assessed by Mann–Whitney U test) between "western diet" and "control diet" groups, regardless of sex group, were reported with a, b, c significantly different; $p < 0.05$, $p < 0.01$ or $p < 0.001$, respectively. Values of *p*-values (assessed by Mann–Whitney U test) between "female" and "male" groups considering each diet group, were reported with *, $p < 0.05$, **, $p < 0.01$ significantly different. PUFA: Polyunsaturated fatty acid; LA: Linoleic acid; ALA: alpha-Linolenic acid. SAT: saturated fatty acids; MCSAT: medium chain saturated fatty acids (C8:0 to C14:0); MUFA: monounsaturated fatty acid; OA: oleic acid ; LC-PUFA: Long-Chain PUFA (polyunsaturated fatty acid that contains at least 16 carbons).

Supplementary Figure

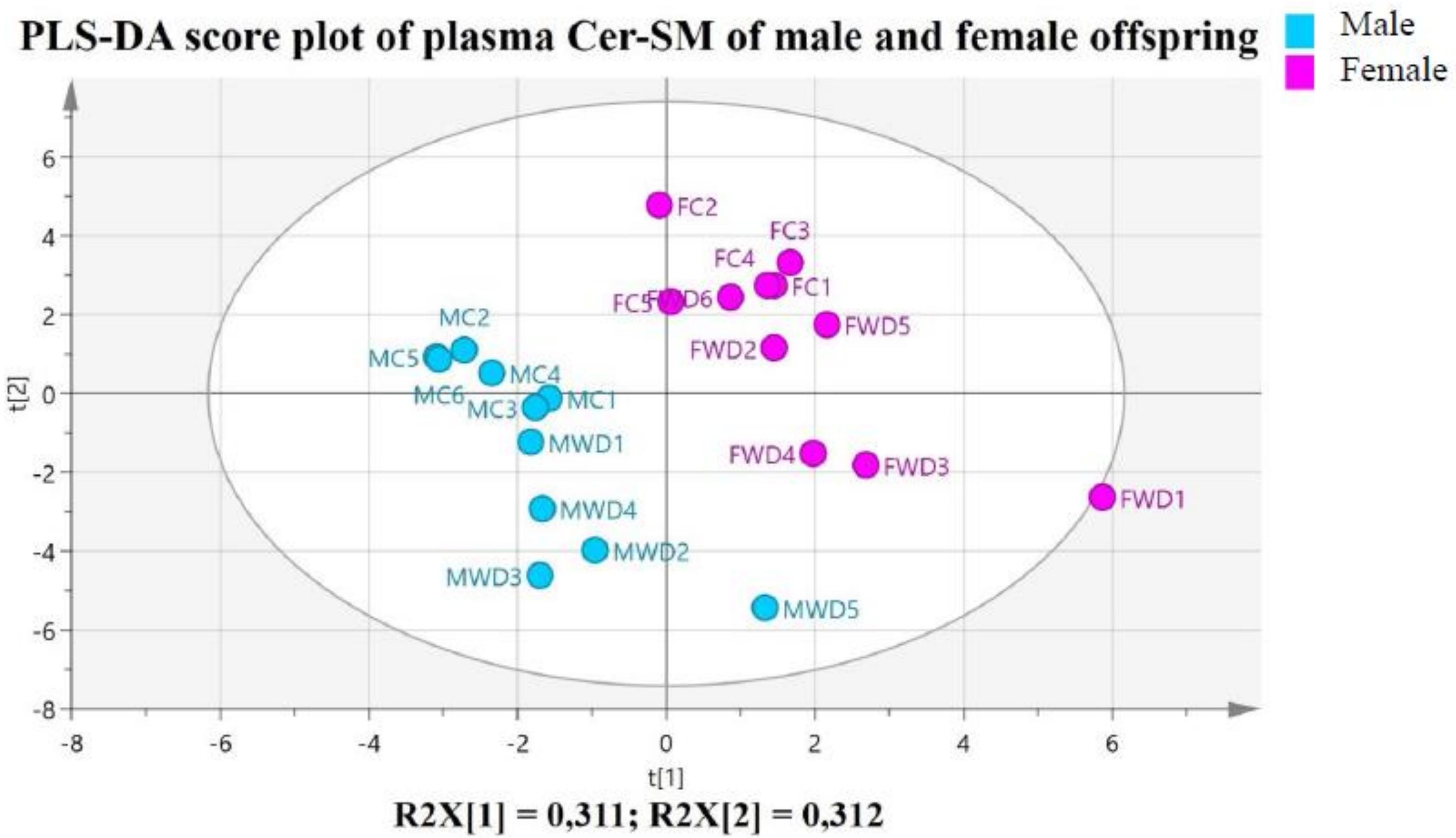

Figure 1. PLS-DA score plot for 25 data points (SLs), reveals natural clusters for all the male (MC, MWD) and female (FC, FWD) 25 days old-offspring.
